# Faraday effects emerging from the optical magnetic field

**DOI:** 10.1038/s41598-025-24492-9

**Published:** 2025-11-19

**Authors:** Benjamin Assouline, Amir Capua

**Affiliations:** Institute of Electrical Engineering and Applied Physics, The Hebrew University of Jerusalem, 91904 Jerusalem, Israel

**Keywords:** Optics and photonics, Physics

## Abstract

The Faraday effect (FE) is commonly attributed to the electrical component of optical radiation. Recently, we reported on an inverse-FE (IFE) that emerges from the Zeeman energy arising from the optical magnetic field. Here, we show that the magnetic component of light reproduces additional signatures observed experimentally in the IFE. Consequently, we show that the magnetic component of light also contributes to the reciprocal, direct FE. Calculating the Verdet constant for the well-studied Terbium-Gallium-Garnet, we find that it accounts for $\sim 17\%$ of the measured value at 800 mm. The Verdet constants derived for the FE and IFE are found to be different, consistent with the breakdown of reciprocity between the two effects in the nonequilibrium ultrafast timescales as reported previously. Our findings highlight the role of the optical magnetic field in the interaction between light and spins, in addition to the primary effects that stem from the electrical field.

**Supplementary Information:**

The online version contains supplementary material available at 10.1038/s41598-025-24492-9.

## Introduction

The Faraday effect (FE), first observed in 1845, provided the earliest experimental evidence of the interaction between optical radiation and magnetism. In this phenomenon, the application of a static magnetic field induces circular birefringence in a material leading to a rotation of the polarization plane of a propagating optical beam. The discovery of the FE marked a foundational milestone in the development of magneto-optics, a field that remains actively investigated to date. A notable contemporary example is the all-optical helicity-dependent switching (AO-HDS) phenomenon, which has attracted considerable attention in recent years^1–4^.

In AO-HDS, ultrashort circularly polarized (CP) optical pulses are used to control the magnetization order parameter. The effect was demonstrated in the single- and multi- pulse domains^5,6^, and recently even under continuous wave (CW) illumination^7^. The experimental evidence showed that the effect depends on a variety of parameters including magnetic structure^8,9^, material composition^10,11^, and laser parameters^12,13^. Consequently, numerous thermal^14–17^, photomagnetic^18,19^, and optomagnetic^13^ mechanisms have been discovered, which originate from the optical electrical field. The thermal mechanisms involve the interaction between electrons, spins, and a phonon bath, and include the ultrafast demagnetization driven by electron heating and absorption of the laser radiation as explored by Kampfrath et al.^20–22^. In the photomagnetic and optomagnetic mechanisms, the optical light irradiation induces a magnetic transition. These mechanisms include, for example, the optical spin transfer torque^23,24^ and optical spin–orbit torque^25–28^ that generate spin polarized currents that exert a torque on the magnetization.

Among the coherent optomagnetic mechanisms, the inverse Faraday effect (IFE), was found to play a vital role in many studies^29–33^. In 1966, Pershan et al.^34^ developed a phenomenological formulation of the IFE, where a magnetic moment is induced by the optical electrical field in a nonlinear process. According to this theory, the same second-order magneto-optical susceptibility of the FE, ${\chi }_{NL}^{(2)}$, is responsible also for the IFE. Consequently, the induced magnetization in the IFE depends on the optical intensity, $I$, specifically, on the intensity difference between the right and left CP (RCP and LCP, respectively) components of the beam,* I*_*RCP*_−* I*_*LCP*_^34–37^. Recently, we showed that beyond the well-established effects that originate from the optical electrical field, also the magnetic field can contribute to the IFE^38^. Namely, according to the Landau-Lifshitz-Gilbert (LLG) equation, a magnetic torque emerges from the Zeeman energy of a CP optical magnetic field. This process was characterized by an interaction strength parameter, $\eta$, which was determined from the ratio between the optical cycle and Gilbert relaxation times according to $\eta =\alpha \gamma {H}_{opt}/{f}_{opt}$ where ${f}_{opt}$, ${H}_{opt}$, and $\alpha$ are the optical frequency, magnetic field amplitude, and the Gilbert damping, respectively, and $\gamma$ is the gyromagnetic ratio. For typical experiments using femtosecond pulses at 800 nm^1,13,19,39^, $\eta$ is in the range of $\sim {10}^{-4}$. Overall, in comparison to the empirical data^31,38^, the calculated torque was found to be sizeable yet insufficient to solely account for the measured values, highlighting the primary contribution of the electric field^20–24^.

These calculations rely on two assumptions: A) The first is that the macrospin approximation pertains. Under this approximation, the magnetization, $\overrightarrow{M}$, is spatially uniform. When this assumption breaks down, $\overrightarrow{M}$ nucleates into a texture and the LLG equation applies to the local domains in which the optical torque is uniform over all spins. B) The second assumption is that the losses are transverse such that $\left|\overrightarrow{M}\right|$ is preserved. In the LLG equation the transverse losses are manifested by $\alpha$, representing a viscous dissipation mechanism. These losses depend on the interaction timescale. When the timescale of the dynamics become comparable to the spin–orbit coupling timescales, the transfer of spin angular momentum to the lattice might be affected^4^. Additionally, the losses are governed by the spin diffusion length, which depends on temperature^40–42^. Therefore, the optically induced thermal heating^14–17^ also influences the loss dynamics. In this case, the Landau-Lifshitz-Bloch (LLB) equation^43^ describes the dynamics more accurately, as explored by Korniienko et al.^44^. In the quantitative experimental study of the optically induced torque in Ref.^31^, which served as a benchmark for comparison with the outcome of the LLG model^38^, the applied optical fluence resulted in a maximum demagnetization of 4%. Namely, the assumption that $\left|\overrightarrow{M}\right|$ is conserved was overall valid.

Here, we show the relevance of the optical magnetic field also to the FE. We start by showing the properties of the optically induced torque that reproduce behaviors seen empirically in the IFE. These include the linear dependence on the optical fluence^5,6,13,26,37^ and more generally on ${I}_{RCP}-{I}_{LCP}$^34–37^, the dependence (independence) of the longitudinal (transverse) torque on $\alpha$^31^, and the build-up of the torque in the multi-pulse and CW regimes. These similarities suggest that the contribution of the optical magnetic field may also be relevant to the reciprocal, direct FE. In this case, we find that the externally applied static magnetic field breaks the symmetry between the interactions with LCP and RCP radiation. By considering the linear magnetic susceptibilities of RCP and LCP radiation stemming from the LLG equation in the highly off-resonant limit, we derive an analytical expression for the Verdet constant. We find it to be wavelength independent, accounting for 17.5% of the measured value for Terbium-Gallium-Garnet (TGG) at 800 nm^37^, and up to 75% at 1.3 µm^45^. Lastly, we show that the Verdet constants resulting from the LLG equation for the FE and IFE are fundamentally different. This result reproduces the well-known breakdown of reciprocity between the FE and IFE resulting from the electrical field when reaching the out-of-equilibrium ultrafast timescales^32,37,46–48^.

## Results

### Implications of the optical magnetic field to the IFE

**Fig. 1:**
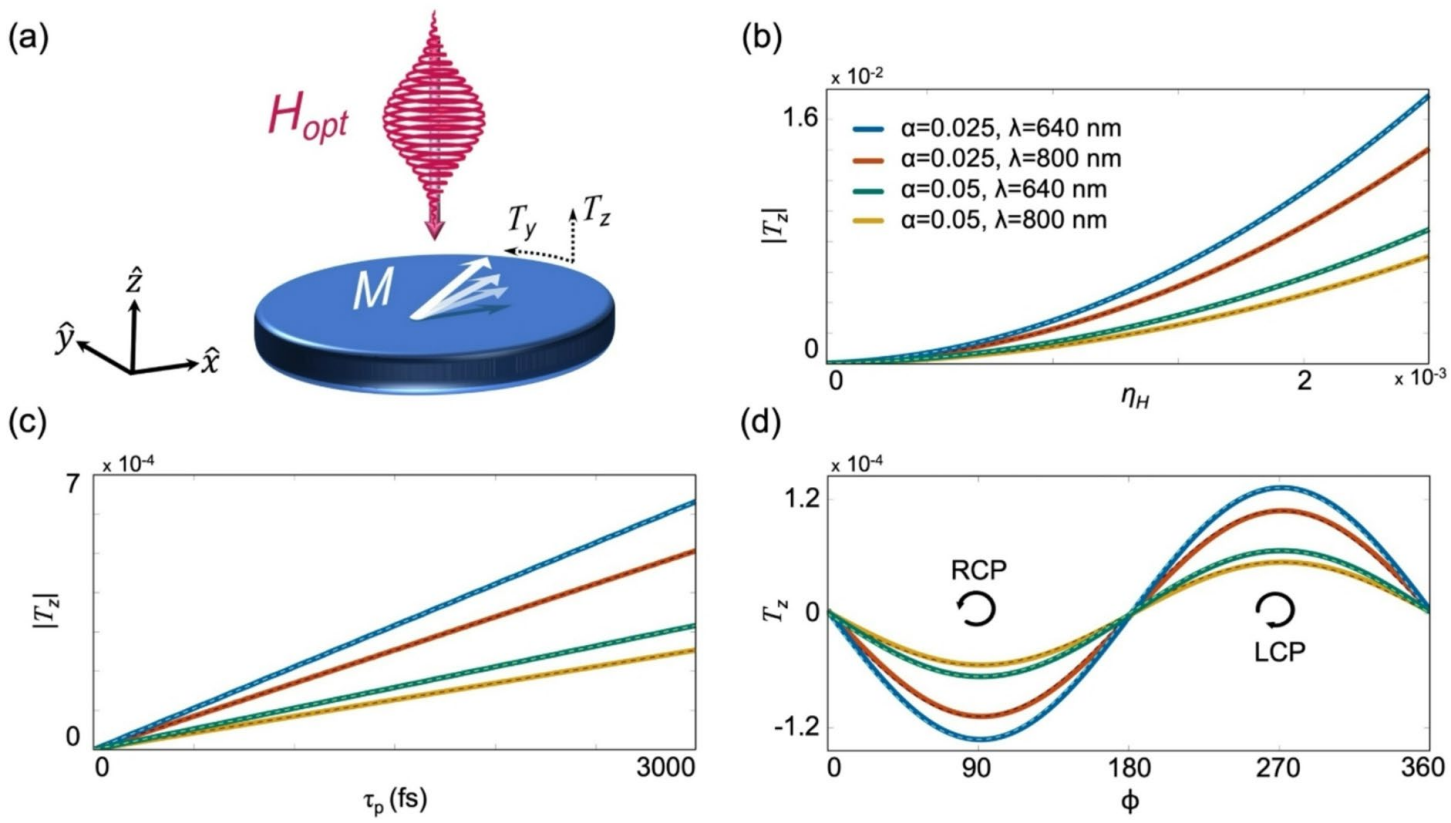
Dependence of the torque on the pulse parameters. (**a**) Schematic illustration of the normalized longitudinal and transverse torques ${T}_{z}$ and ${T}_{y}$, respectively, induced by the optical pulse. (**b**) $|{T}_{z}|$ after the application of an RCP Gaussian magnetic pulse as a function of ${\eta }_{H}$, which is varied by sweeping ${H}_{peak}$ for each $\alpha =0.025, 0.05$ and $\lambda =800, 640\text{ nm}$ value. ${\tau }_{p}=540\text{ fsec}$. Dashed lines correspond to quadratic fits. (**c**) Dependence of $|{T}_{z}|$ on ${\tau }_{p}$, under ${\eta }_{H}=2\times {10}^{-4}$. Dashed lines correspond to linear fits. (**d**) ${T}_{z}$ as a function of $\phi$, under ${\eta }_{H}=2\times 1{0}^{-4}$ and ${\tau }_{p}=540\text{ fsec}$. Dashed lines correspond to $-sin(\phi )$ fits. Panels (c) and (d) follow the color code of (b).

To show the relevance of the magnetic component to the FE, we begin by characterizing the optically induced torque and show that it displays similarities with previous experimental reports on the IFE. We start by examining the effect of a single optical pulse on the macroscopic $\overrightarrow{M}$ as illustrated in Fig. 1a. To this end, we numerically integrate the LLG equation in which the losses are incorporated in the Landau–Lifshitz form^38,49^:

1 $$\frac{{d\vec{M}}}{dt} = - \gamma^{\prime}\left( {\vec{M} \times \vec{H}_{opt} + \frac{\alpha }{{M_{s} }}\vec{M} \times \vec{M} \times \vec{H}_{opt} } \right).$$

Here, ${M}_{s}$ is the saturation magnetization and ${\gamma }^{\prime}=\gamma {\mu }_{0}/(1+{\alpha }^{2})$, where ${\mu }_{0}$ is the magnetic permeability. We apply a right circularly-polarized (RCP) Gaussian pulse of the form $$\vec{H}_{opt} \left( t \right) = H_{peak} \left( {\begin{array}{*{20}c} {\cos \left( {2\pi f_{opt} t} \right)} \\ {\cos \left( {2\pi f_{opt} t - \phi } \right)} \\ 0 \\ \end{array} } \right)e^{{ - \frac{{(t - t_{peak} )^{2} }}{{2\tau_{p}^{2} }}}}$$ where $\phi =90^\circ$. The full-width at half maximum (FWHM) of the intensity is $2\sqrt{ln(2)}{\tau }_{p}$ and the peak amplitude ${H}_{peak}$ is reached at ${t}_{peak}$. Throughout the simulations, $\overrightarrow{M}$ is initialized in $\widehat{x}$ and ${M}_{s}=3\times 1{0}^{5} A/m$ which is typical of Co-based films used experimentally^31^.

We first examine the dependence of the longitudinal torque on the optical fluence, $F$, where the latter is proportional to the product of the intensity and pulse duration: $F \propto H_{peak}^{2} \tau_{p}$. Following the interaction, the accumulated torque results in a tilting of $\overrightarrow{M}$. The longitudinal tilting is determined from the induced $\widehat{z}$ component of $\overrightarrow{M}$ after the interaction is completed, and is represented in normalized units, ${T}_{z}={M}_{z}/{M}_{s}$. Figure 1b presents ${T}_{z}$ as a function of $\eta$ for different typical experimental conditions of $\alpha$ and wavelength, $\lambda$^1,7,13,39^. $\eta$ is varied by sweeping over the relevant range of ${H}_{peak}$ values for each combination of $\alpha$ and $\lambda$. For clarity, we use the parameter ${\eta }_{H}$ to indicate the sweeping over ${H}_{peak}$. The figure readily shows that ${T}_{z}$ is quadratic in ${\eta }_{H}$, namely, ${T}_{z}$ is linear in the optical intensity. Figure 1c illustrates the dependence of ${T}_{z}$ on ${\tau }_{p}$ for a constant ${\eta }_{H}$ illustrating that ${T}_{z}$ is linear in ${\tau }_{p}$ in agreement with the trend reported experimentally in Refs.^5,6^. This behavior indicates that the optically induced torque builds up with each optical cycle. Since ${T}_{z}$ scales with ${H}_{peak}^{2}$ and ${\tau }_{p}$, it also scales with the fluence $F$. A linear dependence of the torque on $F$ was also reported experimentally^5,6,13,26,37^ and was attributed to the non-linear susceptibility ${\chi }_{NL}^{(2)}$^34,35,37^. Following a detailed analytical derivation (see Supplemental Note 1), we find that $T_{z} = \frac{{\gamma^{2} }}{2\sqrt \pi }\frac{\alpha }{{f_{opt} }}H_{peak}^{2} \tau_{p} \propto F\alpha /f_{opt}$ (throughout the work we use ${\gamma }^{\prime}\approx \gamma$). This relation also shows that the torque is enhanced with $\alpha$ and decreases with ${f}_{opt}$. Interestingly, the AO-HDS was demonstrated in a variety of multi-layered material systems that consist of heavy metals such as Pt and Pd which possess large $\alpha$^24,26,31,39^. We point out that beyond the macrospin approximation, a spatial distribution could also affect the interaction. Such spatial dependence was recently investigated numerically by Zhang et al. in Ref.^50^, where the optical profile and spin texture were calculated, enabling ultrafast excitation and control of the helicity of skyrmions using CP light.

The dependence ${T}_{z}\propto F$ implies that the effect should be prominent for higher powers, where the pulse heating is higher, which may lead to electron heating due to absorption as explored by Kampfrath et al.^20–22^. Maehrlein et al.^20^ demonstrated that angular momentum transfer in yttrium iron garnet occurs in two stages that are characterized by distinct time constants: rapid spin-phonon energy equilibration within 1 picosecond, followed by angular momentum transfer to the lattice over 100 ns. Furthermore, Rouzegar et al.^22^ showed that ultrafast demagnetization and terahertz spin transport, previously considered distinct phenomena, share a common origin driven by a generalized spin voltage in a ferromagnet. Interestingly, in Ref.^21^ Chekhov et al. reported that the demagnetization does not depend on the wavelength and can equally take place with optical and terahertz (THz) excitations. In this case, the torque induced by the optical magnetic field may be described in more detail by the LLB equation^43^, where also longitudinal relaxation takes place. Such approach was explored by Korniienko et al.^44^, where the interaction with intense ultrashort THz pulses was studied in the framework of the LLB equation and a two temperature model coupling electrons and phonons. In Supplemental Notes 2 and 3 we include the anisotropy field and the longitudinal relaxation term, respectively, where it is seen that they have a negligible effect on the optically induced torque for the typical experimental settings we consider.

For a general polarization state, ${T}_{z}$ is described by the difference between the RCP and LCP fluences. This is illustrated in Fig. 1d by plotting ${T}_{z}$ as a function of the polarization state $\phi$. It is readily seen that ${T}_{z}$ vanishes for linearly polarized (LP) beams ($\phi =0^\circ ,180^\circ$) whereas for CP beams ($\phi =90^\circ ,270^\circ$) it is maximal, which is typical of AO-HDS^1,6,26,51^. For a general $\phi$, ${T}_{z}\propto -\mathit{sin}\left(\phi \right)$ (see Supplemental Note 1) which is proportional to ${I}_{RCP}-{I}_{LCP}$ (see Supplemental Note 4). Hence, ${T}_{z}$ is given by:

2 $$T_{z} = \frac{{\gamma^{2} }}{2\sqrt \pi c} \cdot \frac{\alpha }{{f_{opt} }}\left( {I_{RCP} - I_{LCP} } \right)\tau_{p} ,$$

where $c$ is the speed of light. Equation (2) is valid for small angle dynamics corresponding to small values of $\eta$ (see Supplemental Note 1).

The dependence of ${T}_{z}$ on ${I}_{RCP}-{I}_{LCP}$ also appears in Pershan’s phenomenological description of the IFE which was derived from the free energy of the crystal in the presence of the electrical component of the radiation, $\overrightarrow{E}$. Accordingly, ${M}_{z}\propto {\chi }_{NL}^{(2)}\left|\overrightarrow{E}\times {\overrightarrow{E}}^{*}\right|\propto {I}_{RCP}-{I}_{LCP}$^34,37^. The potential function derived from Pershan’s Hamiltonian assumes a slowly varying optical intensity as compared to the thermal relaxation times of the system^52^. This assumption does not hold in the ultrashort timescales and several studies^37,46,47,52,53^ showed that the standard dependence on $\overrightarrow{E}\times {\overrightarrow{E}}^{*}$ is incomplete in this limit. Reid^46^, Popova^52^, and Battiato et al.^32^ showed that on the subpicosecond timescales a stimulated magneto-Raman scattering process takes place which is known as the ultrafast-IFE.

**Fig. 2:**
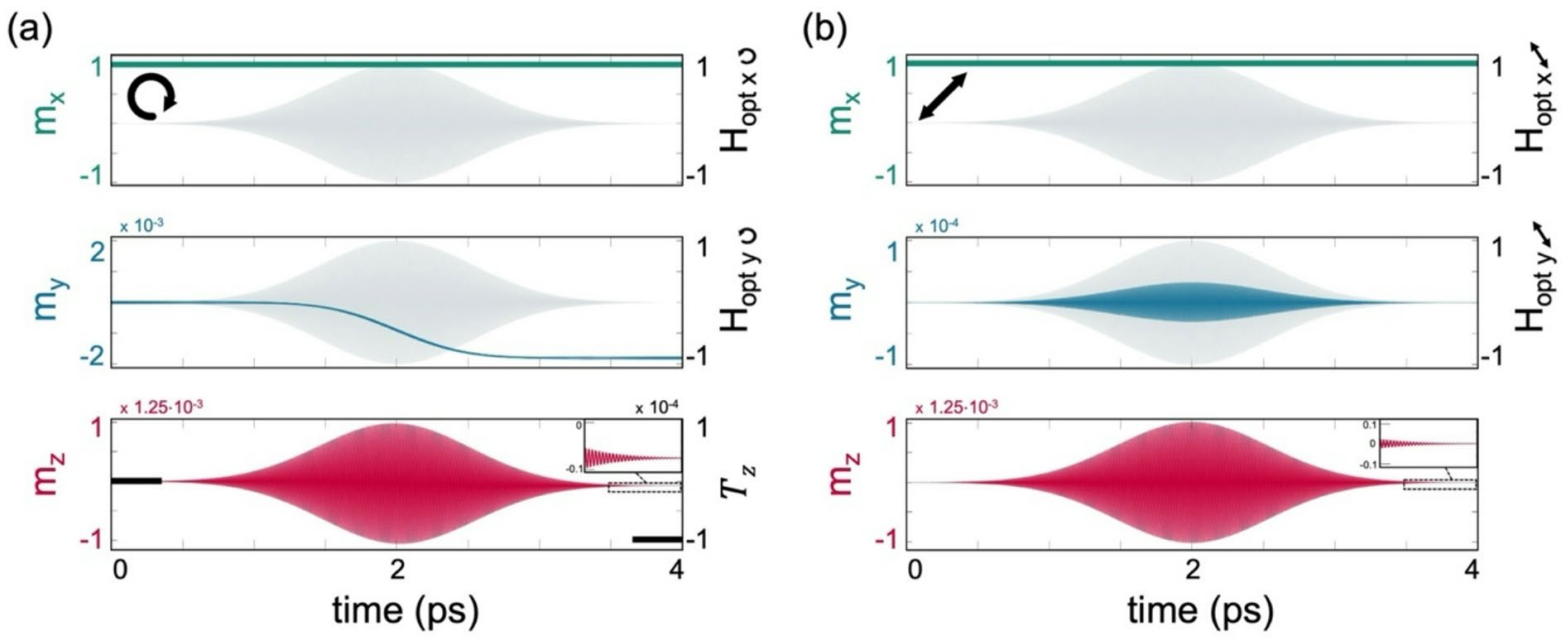
CP and LP single pulse dynamics. (**a**) Temporal evolution of $\overrightarrow{m}=\overrightarrow{M}/{M}_{s}$ induced by an RCP Gaussian pulse under ${\eta }_{H}=2\times 1{0}^{-4}$ and ${\tau }_{p}=540 \text{ fsec}, {t}_{peak}=2\text{ psec}$. Top and middle panels depict the temporal evolution of the $x$ and $y$ components of $\overrightarrow{m}$ and ${\overrightarrow{H}}_{opt}$ in normalized units, and the bottom panel depicts ${m}_{z}$. Inset: zoomed in dynamics of ${m}_{z}$ following the pulse. (**b**) Dynamics under LP pulse. $\alpha =0.025$ and λ = 800 nm in (**a**) and (**b**).

The calculated transverse magnetization tilting also reproduces trends observed experimentally. It is represented by ${T}_{y}={M}_{y}/{M}_{S}$ following the interaction. To illustrate this point, we examine the temporal evolution of $\overrightarrow{M}$. Figure 2a presents the normalized $\overrightarrow{m}(t)=\overrightarrow{M}/{M}_{S}$, for $\alpha =0.025$, λ = 800 nm, ${\eta }_{H}=2\times {10}^{-4}$, and ${\tau }_{p}=540\text{ fsec}$ resulting in ${T}_{y} \sim -2\times {10}^{-3}$. Following the same numerical analysis and analytical derivation, we find that ${T}_{y}$ is independent of $\alpha$ such that ${T}_{y}\propto F/{f}_{opt}$ (see Supplemental Note 1). The dependence (independence) of ${T}_{z}$ (${T}_{y}$) on $\alpha$ was also observed experimentally by Choi et al.^31^ using time-domain vectorial torque measurements, where $\alpha$ was varied by changing the metallic capping layer in ferromagnet (FM)/metallic bilayers.

Interestingly, in Ref.^30^, Ali et al. demonstrated that an effective IFE magnetic field can be induced even under an LP beam. The effect arises from the transfer of orbital angular momentum from a beam with a helical wavefront, where each of the orthogonal Laguerre-Gaussian modes composing the helical wavefront transfers its well-defined photon orbital angular momentum to the plasma. This result shows that angular momentum could also be transferred by means other than the circular polarization of light and stimulates the investigation of LP beams in our case. For comparison, in Fig. 2b we examine the temporal evolution of $\overrightarrow{M}$ driven by a single LP ${\overrightarrow{H}}_{opt}$ pulse ($\phi =0^\circ$), under the same conditions used in Fig. 2a. It is readily seen that although at the end of the interaction the net induced torque is zero, $\overrightarrow{M}$ undergoes a non-trivial dynamical evolution. Further investigation of the dependence of the LP case on the pulse power, duration, and polarization direction is presented in Supplemental Note 5, illustrating that the polarization direction affects the dynamical evolution while the resultant torque remains zero.

**Fig. 3:**
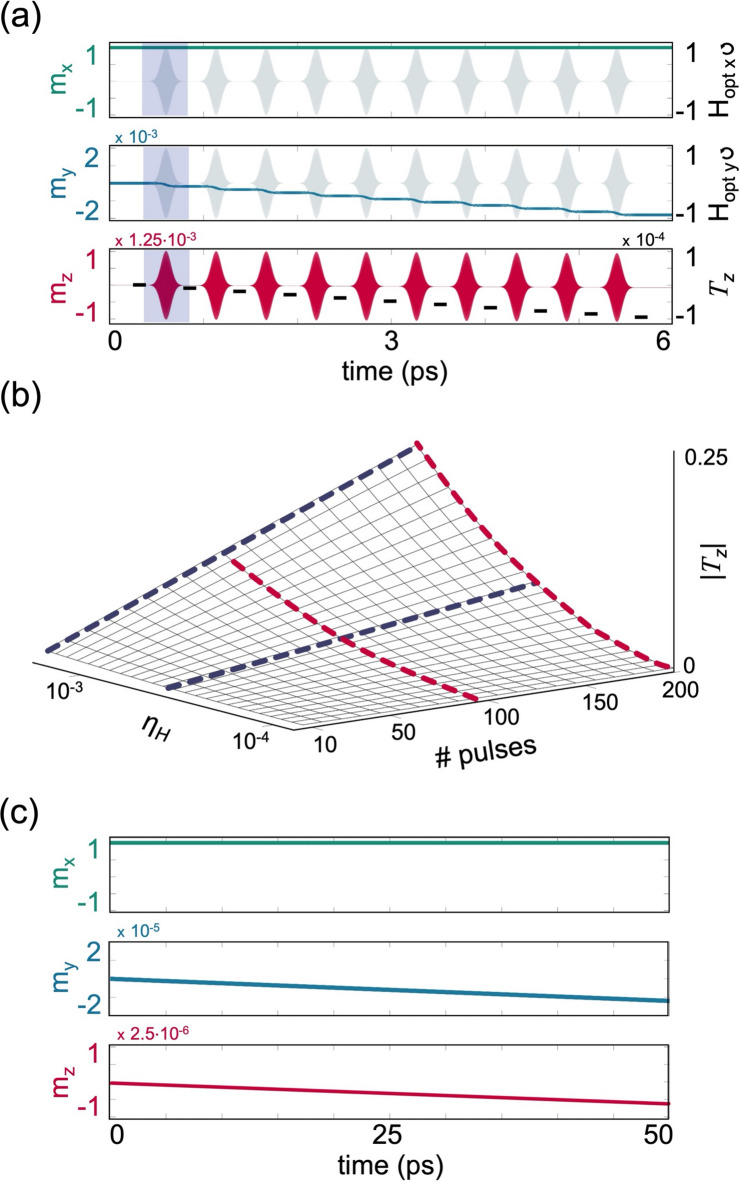
Multi-pulse and CW regimes. (**a**) Temporal evolution of $\overrightarrow{m}$ under ${\eta }_{H}=2\times 1{0}^{-4}$ and ${\tau }_{p}=54 \text{ fsec}$, induced by 10 RCP Gaussian magnetic pulses. Top and middle panels depict the temporal evolution of the $x$ and $y$ components of $\overrightarrow{m}$ and ${\overrightarrow{H}}_{opt}$ in normalized units, and the bottom panel depicts ${m}_{z}$. For visibility, brown dashed lines representing ${T}_{z}$ induced by each pulse are added and the interaction with the first pulse is highlighted. (**b**) Normalized $|{T}_{z}|$ after the application of RCP pulses as a function of ${\eta }_{H}$ and the number of pulses, where ${\tau }_{p}=54 \text{ fsec}$ for each pulse. Red and blue curves correspond to quadratic and linear fits, respectively, and are guides to the eye. (**c**) Temporal evolution of $\overrightarrow{m}$ induced by a CW RCP magnetic field under $\eta ={10}^{-7}$. Top, middle, and bottom panels depict the evolution of the $x$, $y$, and $z$ components of $\overrightarrow{m}$, respectively. In (**a**) to (**c**), $\overrightarrow{m}$ is initialized in $\widehat{x}$, $\alpha =0.025$, and λ = 800 nm as in Fig. 2.

The torque induced by a single pulse can be equivalently achieved by applying multiple pulses whose total fluence equals that of the original pulse. Figure 3a presents the temporal response of $\overrightarrow{m}$ to $10$ identical ${\overrightarrow{H}}_{opt}$ pulses, applied as in Fig. 2a, except that each pulse has one tenth of the duration ${\tau }_{p}$, and an arbitrary carrier phase. It is seen that following the entire interaction, the accumulated torque is equal to the torque induced by the original single pulse of Fig. 2a and is independent of the relative carrier phases. Figure 3b presents ${T}_{z}$ induced by multiple RCP ${\overrightarrow{H}}_{opt}$ pulses as a function of ${\eta }_{H}$ and the number of applied pulses. It is seen that ${T}_{z}$ is linear in the number of pulses and quadratic in ${\eta }_{H}$. In this general case, ${T}_{z}=\frac{1}{2\sqrt{\pi }\alpha }\frac{\#pulses\times {\tau }_{p}}{{t}_{cycle}}{\eta }^{2}$ (see Supplemental Note 6) such that the optically-induced torque builds up with each applied pulse as also reported experimentally^5,6,51^. The total torque can be induced either by a single pulse or by multiple pulses which further demonstrates that ${\rm T}_{z}$ scales with the accumulated exposure time.

The dependence on the exposure time suggests that the effect may be also relevant for longer pulses reaching the CW limit as reported recently by Stenning et al. ^7^. The dynamics induced by a rectangular quasi-CW pulse are depicted schematically in Fig. 3c by introducing an RCP CW beam, ${\overrightarrow{H}}_{opt CW}\left(t\right)$, at 800 nm for a duration of ${t}_{CW}=50 \text{nsec}$. In the simulation, ${\overrightarrow{H}}_{opt CW}$ corresponds to a $5 \text{ mW}$ laser beam that is focused to a diameter of $500\text{ nm}$. Under these settings, ${H}_{peak}$ was $\sim 10 \text{ mT}$ for which $\eta \sim {10}^{-7}$ with $\alpha =0.025$ as in Fig. 2. Figure 3c reveals similar features seen in the single- and multi-pulse cases with ${T}_{z} = \frac{1}{2\pi \alpha }\frac{{t_{CW} }}{{t_{cycle} }}\eta^{2}$ (see Supplemental Note 7). In Ref. ^7^ the magnetization reversal was induced by a 633 nm CW beam in Py nanomagnets. A deterministic low power switching was demonstrated over long exposure times of ${t}_{CW} \sim$ 1 sec where the laser power was $\sim$ 2.5–5 mW focused to a spotsize of 580 nm diameter. Under these settings, the magnetic field amplitude is $\sim$ 6.5–10 mT. Considering $\alpha =0.015$, which is typical for Py, the corresponding $\eta$ is $\sim 4-6\times {10}^{-8}$. Using the expression above for ${T}_{z}$, we find that in order to fully switch $\overrightarrow{M}$ and reach ${T}_{z}=1$, the required ${t}_{CW}$ is $\sim$ 0.1 sec which is of the same order of magnitude of the reported exposure times. We remark that the experiment was conducted with LP CW beams in highly magnetically anisotropic nanomagnets and the effect was attributed to an asymmetric absorption.

### Implications to the FE

**Fig. 4:**
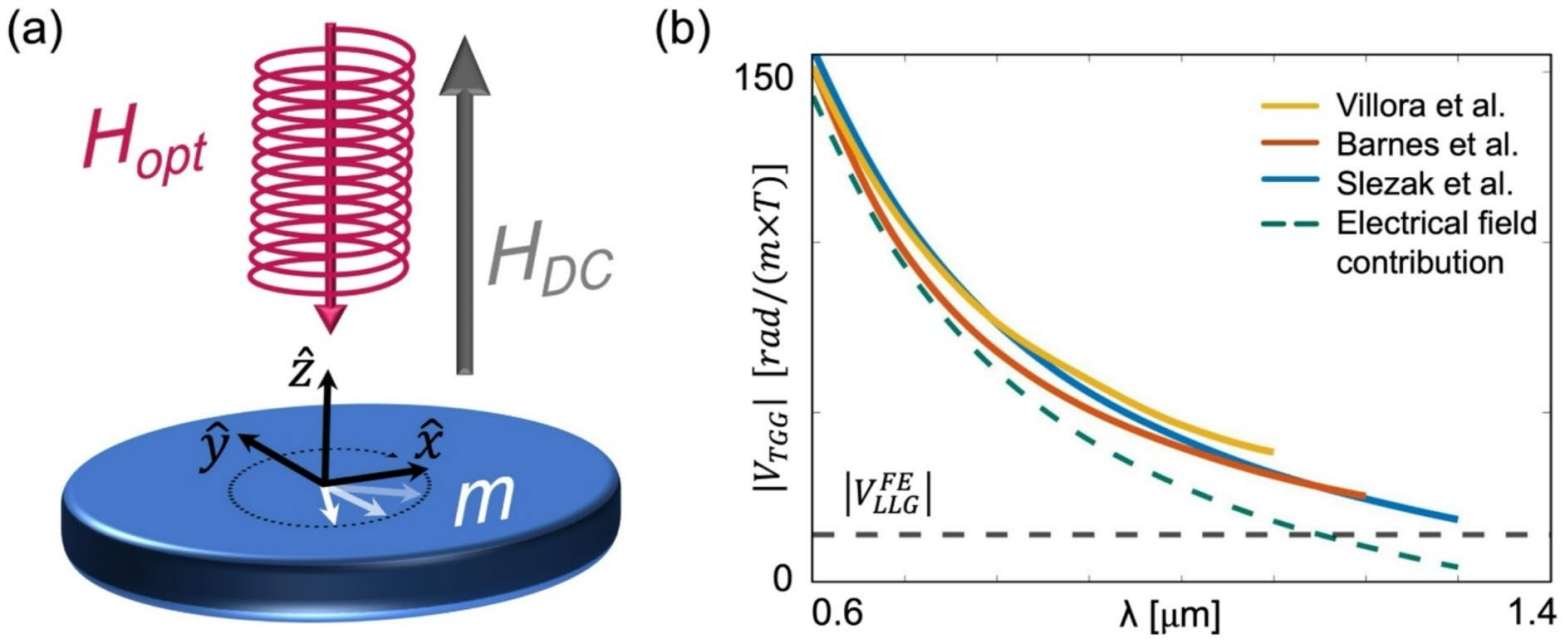
FE stemming from the optical magnetic field. (**a**) Schematic illustration of the steady dynamics induced by a CP CW optical beam in the presence of an external static field. (**b**) Comparison between the calculated ${V}_{LLG}^{FE}$ and the Verdet constants from Refs.^45,54,55^. Empirical data adopted from Ref.^45^.

The relevance of the LLG equation to the IFE and to the CW regime suggests that the LLG framework may also be related to the direct FE. In the FE, an external magnetic field is applied which breaks the symmetry between LCP and RCP radiation while the interaction occurs in steady state, as depicted in Fig. 4a. Furthermore, in contrast to the case of the IFE, $\overrightarrow{M}$ is not spontaneous, rather, it is induced by the static magnetic field. To evaluate the FE stemming from the optical magnetic field, we calculate the Verdet constant, $V,$ from the circular birefringence by calculating the magnetic susceptibilities for RCP and LCP states, ${\chi }_{RCP}$ and ${\chi }_{LCP}$, respectively. From the linearized LLG equation (see Supplemental Note 8):

3 $$\chi_{RCP} = \frac{{ - \gamma \mu_{0} M_{S} }}{{\omega - \gamma \mu_{0} H_{DC} - j\omega \alpha }} , \chi_{LCP} = \frac{{\gamma \mu_{0} M_{S} }}{{\omega + \gamma \mu_{0} H_{DC} + j\omega \alpha }}$$

where ${H}_{DC}$ is the amplitude of the externally applied static field and $\omega$ is the optical angular frequency. The Faraday rotation angle, ${\Theta }_{FE}$, is expressed by the product of the RCP and LCP wavenumber difference,${k}_{RCP}-{k}_{LCP}$, and the optical length, $L$: ${\Theta }_{FE}=\frac{1}{2}\left({k}_{RCP}-{k}_{LCP}\right)L$. Using ${k}_{RCP/LCP}=\omega \sqrt{{\epsilon }_{r}(1+{\chi }_{RCP/LCP}) }/c$, for highly off-resonance conditions we obtain:

4 $$\begin{array}{*{20}c} {{\Theta }_{LLG}^{FE} = - \frac{1}{2}\frac{{\gamma \mu_{0} M_{S} }}{{1 + \alpha^{2} }}\frac{{\sqrt {\varepsilon_{r} } }}{c}L,} \\ \end{array}$$

where ${\epsilon }_{r}$ is the relative electrical permittivity. Using ${\Theta }_{FE}=V{\mu }_{0}{H}_{DC}L$ and substituting ${M}_{S}={\mu }_{0}{\chi }_{DC}{H}_{DC}$ with ${\chi }_{DC}$ being the DC magnetic susceptibility, we obtain:

5 $$V_{LLG}^{FE} = - \frac{1}{2}\frac{{\sqrt {\varepsilon_{r} } }}{{1 + \alpha^{2} }}\frac{\gamma }{c}\mu_{0} \chi_{DC} ,$$

where the notation ${V}_{LLG}^{FE}$ indicates $V$ that is calculated from the LLG equation. ${V}_{LLG}^{FE}$ is wavelength-independent aside from the dispersion of ${\epsilon }_{r}$.

To assess the calculation, we examine the well-studied paramagnetic Terbium-Gallium-Garnet (TGG) crystal that possesses a high magnetic susceptibility and is commonly used in FE-based optical components. Taking ${\chi }_{DC}=2\times {10}^{4}\frac{A}{m\times T}$ and ${\epsilon }_{r}=4$ of TGG, we obtain ${V}_{LLG}^{FE}=-14\frac{rad}{m\times T}$ while the measured ${V}_{TGG}$ at 800 nm is ${V}_{TGG}=-80\frac{rad}{m\times T}$^56,57^. ${V}_{LLG}^{FE}$ accounts for a significant yet partial $17.5\%$ contribution to ${V}_{TGG}$. The experimental observations show that in general $V$ is inversely proportional to $\lambda$. According to Becquerel’s classical theory of the FE^58^, a ${\lambda }^{-1}$ dependence arises from the circular trajectory of the charges subjected to the CP electrical field. More recent works showed that $V$ is more accurately described by $V\propto 1/({\lambda }^{2}-{\lambda }_{0}^{2})$ where ${\lambda }_{0}$ is a constant^45,59,60^. Overall, the smallest value of $V$ is expected at the longer wavelengths. We remark that the minimal values of $\left|{V}_{TGG}\right|$ recorded by Villora^54^, Barnes^55^, and Slezak^45^ were $39$, $26.7$, and $18.7 \frac{rad}{m\times T}$ at $\lambda =1.1$, $1.2$, and 1.3 µm, respectively, and are higher than the calculated $\left|{V}_{LLG}^{FE}\right|$. This is illustrated in Fig. 4b by presenting ${V}_{TGG}$ as a function of $\lambda$ as measured by Villora, Barnes, and Slezak together with the lower bound predicted by ${V}_{LLG}^{FE}$. In addition, we present the contribution of the optical electrical field, obtained by subtraction of $\left|{V}_{LLG}^{FE}\right|$, readily showing the significant role of ${\overrightarrow{H}}_{opt}$ at long $\lambda$.

It is possible that in addition to the wavelength independent magnetic contribution, spin–orbit coupling may give rise to a wavelength-dependence of ${V}_{LLG}^{FE}$. We remark that the exchange and anisotropy energies in non-magnetic materials that are subjected to an externally applied field, as in TGG, are generally much weaker as compared to those in ferro- and ferri- magnetic materials. This is due to the absence of spontaneous long-range magnetic ordering as well as a relatively small induced $\overrightarrow{M}$.

## Discussion

According to Pershan’s theory, both the FE and IFE are reciprocal^34^. Namely, the same static $V$ describes both the FE and IFE. In the past two decades, many studies have invalidated this assumption for the highly nonequilibrium ultrashort timescales and showed that reciprocity can only be considered when thermal equilibrium prevails^32,37,46–48^. In order to examine the reciprocity between the two effects as calculated from the LLG equation, we first determine $V$ for the IFE, ${V}_{LLG}^{IFE}$. To this end we express the induced $\overrightarrow{M}$ in the form ${M}_{z}=\frac{{I}_{RCP}-{I}_{LCP}}{2c\pi }\lambda {V}_{LLG}^{IFE}$, adopted from Pershan’s formalism. Comparing this expression with the calculated ${T}_{z}$ of Eq. (2) we obtain:

6 $$V_{LLG}^{IFE} = M_{S} \frac{\sqrt \pi }{c}\frac{\alpha }{{\left( {1 + \alpha^{2} } \right)^{2} }}\gamma^{2} \mu_{0} \tau_{p} .$$

Equations (5) & (6) illustrate that ${V}_{LLG}^{FE}\ne {V}_{LLG}^{IFE}$. Furthermore, ${V}_{LLG}^{FE}$ depends only on the material parameters whereas ${V}_{LLG}^{IFE}$ also depends on the pulse parameters. This is a manifestation of the fact that the two Verdet constants were derived from fundamentally different dynamical regimes. ${V}_{LLG}^{FE}$ was calculated under off-resonant steady state conditions. Therefore, it is proportional to ${\chi }_{DC}$ of the material and only weakly depends on the loss rate, $\alpha$. On the other hand, ${V}_{LLG}^{IFE}$ was derived for a non-adiabatic transition^61,62^ where the electromagnetic radiation and $\overrightarrow{M}$ are not in equilibrium. As such, ${V}_{LLG}^{IFE}$ is proportional to the pulse duration and $\alpha$. Hence, we conclude that also within the LLG framework, the FE and IFE cannot be described by the same $V$ when reaching the ultrashort timescales.

## Summary

In this work we explored the IFE within the framework of the LLG equation and showed that it has implications for the FE. The results display similarities with previous experimental reports on optically induced torques, including the dependence of the torque on $F$ and $\phi$, the dependence (independence) of its longitudinal (transverse) component on $\alpha$, and the emergence of the torque in the single- and multi- pulse regime as well as the CW regime. Additionally, the resulting FE and IFE turn out to be non-reciprocal when reaching the nonequilibrium ultrafast timescales, as reported in previous works. Both effects are found to be sizeable yet incomplete to solely account for the experimental observations and should be considered alongside well-established mechanisms for the FE and IFE that are induced by the electrical field. Future works should elucidate the wavelength dependence of the FE and the possible role of the spin–orbit coupling. Additionally, a more advanced formulation of the LLG equation should be used when approaching the ultrashort timescales where the Gilbert damping may be influenced by ultrafast heating mechanisms^44^. Furthermore, the potential influence of a helical wavefront of the optical magnetic field should also be investigated^30^, where the helicity of light is substituted by the photon orbital angular momentum.

## Supplementary Information

Below is the link to the electronic supplementary material.

Supplementary Material 1

## Data Availability

The data that support the findings of this study are available from the corresponding author upon reasonable request.

## Code availability

The codes used in the theoretical calculations are available from the corresponding author upon reasonable request.
