# Supplementary material for: Faraday effects emerging from the optical magnetic field
Source: Sci Rep. 2025 Nov 19;15:39566. doi: 10.1038/s41598-025-24492-9 (PMC12630884; doi:10.1038/s41598-025-24492-9)
Supplement: Supplementary file 1 — Supplementary Material 1 [file 41598_2025_24492_MOESM1_ESM.pdf]

Faraday effects emerging from the optical magnetic field

Supplemental Material

Benjamin Assouline, Amir Capua*

Institute of Applied Physics, The Hebrew University of Jerusalem, Jerusalem 91904, Israel

*e-mail: amir.capua@mail.huji.ac.il

Contents

Supplemental Note 1: Analytical derivation of T_y and T_z induced by a single pulse 2

Supplemental Note 2: Effect of anisotropy field 9

Supplemental Note 3: Effect of longitudinal relaxation 11

Supplemental Note 4: Dependence of T_z the polarization state 13

Supplemental Note 5: Interaction with linearly polarized pulses 14

Supplemental Note 6: Multi-pulse regime 16

Supplemental Note 7: CW regime 18

Supplemental Note 8: Derivation of V_{LLG}^{FE} and V_{LLG}^{IFE} 19

References 21

Supplemental Note 1: Analytical derivation of T_y and T_z induced by a single pulse

In this Note we analytically derive T_y and T_z for the case of a single pulse. We start by deriving the expression for T_z. We consider the LLG equation:

$$\frac{d\vec{M}}{dt} = -\gamma' \left(\vec{M} \times \vec{H}_{opt} + \frac{\alpha}{M_s} \vec{M} \times \vec{M} \times \vec{H}_{opt} \right), \tag{S1.1}$$

where $\vec{H}_{opt}$ is the optical magnetic field:

$$\vec{H}_{opt}(t) = H_{peak} \begin{pmatrix} cos(2\pi f_{opt} t) \\ cos(2\pi f_{opt} t - \phi) \\ 0 \end{pmatrix} e^{-\frac{(t-t_{peak})^2}{2\tau_p^2}}. \tag{S1.2}$$

The $\hat{z}$ component of Eq. (S1.1) is given by:

$$\frac{dM_z}{dt} = -\gamma' \left(\begin{pmatrix} M_x \\ M_y \end{pmatrix} \times \begin{pmatrix} H_{opt\,x} \\ H_{opt\,y} \end{pmatrix} + \frac{\alpha}{M_s} M_z (H_{opt\,x} M_x + H_{opt\,y} M_y) \right). \tag{S1.3}$$

Taking $\alpha \ll 1$, we approximate:

$$M_z \approx -\gamma' \int \begin{pmatrix} M_x \\ M_y \end{pmatrix} \times \begin{pmatrix} H_{opt\,x} \\ H_{opt\,y} \end{pmatrix} d\tau. \tag{S1.4}$$

We consider initial conditions where $\vec{M}$ is in the $\hat{x}$ direction and calculate M_z at the beginning of the interaction. Therefore Eq. (S1.4) takes the form:

$$M_z = -\gamma' \int M_x H_{opt\,y} \, d\tau. \tag{S1.5}$$

Namely,

$$M_z = -\gamma' \int H_{peak} M_S \, cos(2\pi f_{opt} \tau - \phi) e^{-\frac{(\tau-\tau_{peak})^2}{2\tau_p^2}} d\tau. \tag{S1.6}$$

Considering a slowly-varying envelope of $\vec{H}_{opt}$, Eq. (S1.6) becomes:

$$M_z = -\frac{\gamma' H_{peak} M_S}{2\pi f_{opt}} sin(2\pi f_{opt} t - \phi) e^{-\frac{(t-t_{peak})^2}{2\tau_p^2}}. \tag{S1.7}$$

Substituting Eq. (S1.7) into Eq. (S1.3) and neglecting the fast-rotating terms we obtain:

$$\frac{dM_z}{dt} = -{\gamma'}^2 \alpha M_s \frac{H_{peak}^2}{2\pi f_{opt}} e^{-\frac{(t-t_{peak})^2}{\tau_p^2}} sin(\phi), \tag{S1.8}$$

from which T_z can be calculated:

$$T_z = \frac{1}{M_s} \int \frac{dM_z}{dt} = -\frac{{\gamma'}^2 \alpha}{2\sqrt{\pi} f_{opt}} H_{peak}^2 \tau_p sin(\phi), \tag{S1.9}$$

where the coefficient $\frac{\tau_p}{2\sqrt{\pi}}$ stems from the temporal Gaussian envelope.

To calculate T_y, we examine the $\hat{y}$ component of Eq. (S1.1):

$$\frac{dM_y}{dt} = -\gamma' \left(M_z H_{opt\,x} + \frac{\alpha}{M_s}\left(H_{opt\,y}(M_x^2 + M_z^2) - H_{opt\,x} M_y M_x\right) \right), \qquad (S1.10)$$

where the second term can be neglected since $\alpha \ll 1$, resulting in:

$$\frac{dM_y}{dt} \approx -\gamma' M_z H_{opt\,x}. \qquad (S1.11)$$

Substituting Eq. (S1.7) into Eq. (S1.11) and neglecting the fast-rotating terms gives:

$$\frac{dM_y}{dt} = -\frac{\gamma'^2 H_{peak}^2 M_S}{4\pi f_{opt}} sin(\phi)\, e^{-\frac{(t-t_{peak})^2}{\tau_p^2}}, \qquad (S1.12)$$

from which we obtain:

$$T_y = \frac{1}{M_s}\int \frac{dM_y}{dt} = -\frac{\gamma'^2}{4\sqrt{\pi} f_{opt}} H_{peak}^2 \tau_p sin(\phi). \qquad (S1.13)$$

To validate the analytical solution and determine the conditions under which it applies, we compare it with numerical calculations. In Fig. S1(a) we present T_z as a function of η_H and τ_p for an RCP optical magnetic pulse for $10^{-4} \le \eta \le 2.5 \times 10^{-3}$, $\alpha = 0.025$, and $\lambda = 800\ nm$. The data is presented on a logarithmic scale for visibility. The amplitude of the pulse is $H_{peak} = \eta H_{\eta=1}$, where $H_{\eta=1} = \frac{f_{opt}}{\alpha\gamma'}$ is the magnetic field amplitude required for $\eta = 1$. As in the manuscript, η is varied by sweeping over the relevant range of H_{peak} values for each α and λ and is represented by the parameter η_H. In Fig. S1(b) we present the corresponding analytical solution, which reproduces the numerically integrated calculation.

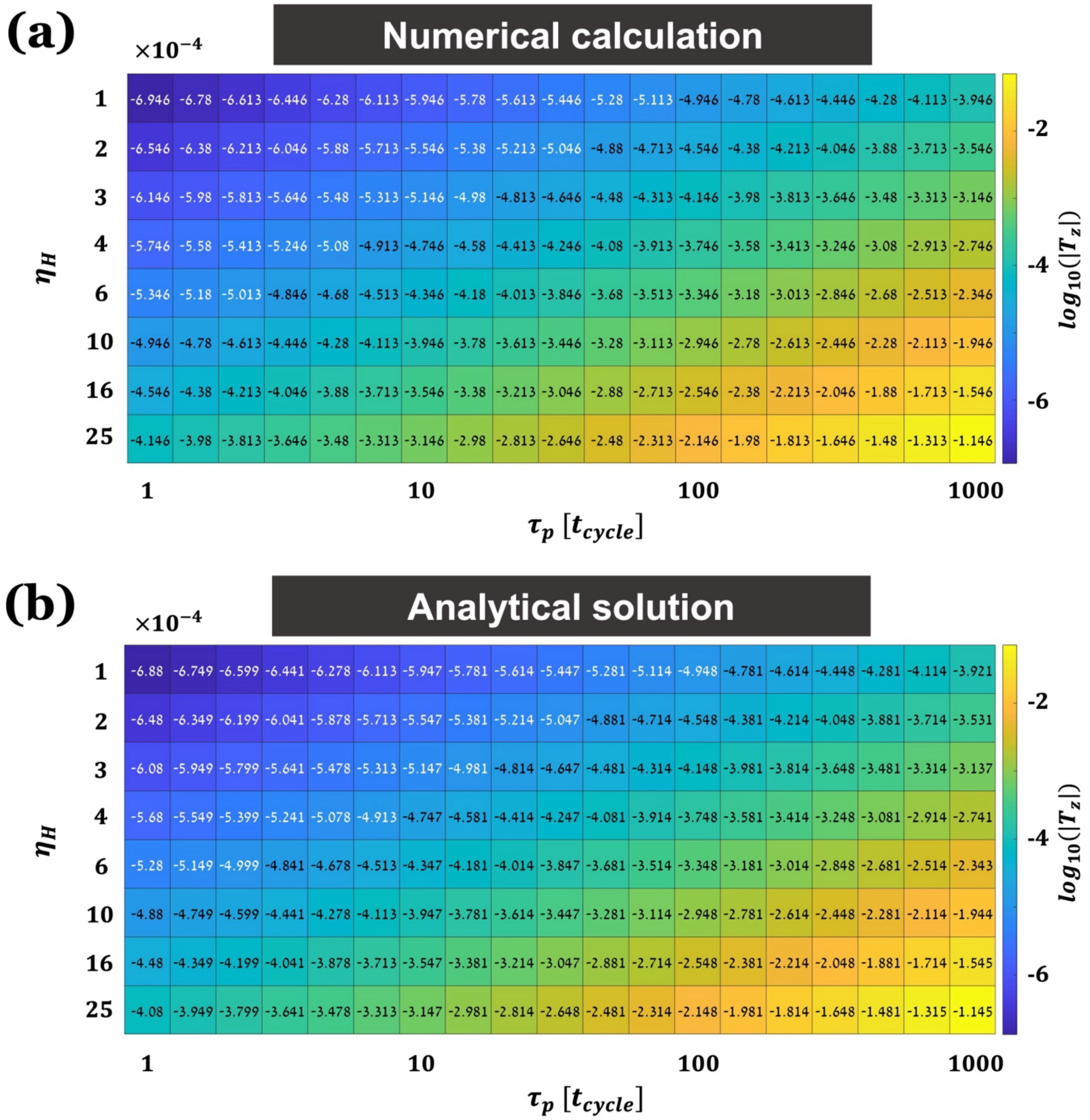

Fig. S1. $log_{10}(|T_z|)$ after the application of an RCP optical magnetic pulse of duration τ_p and amplitude $\eta_H H_{\eta=1}$. $\alpha = 0.025, \lambda = 800\ nm$. $\vec{M}$ is initialized in the $\hat{x}$ direction. (a) Numerical integration of the LLG equation. (b) Analytical solution $\frac{1}{2\sqrt{\pi}\alpha}\frac{\tau_p}{t_{cycle}}\eta^2$. Data is presented on a logarithmic scale for visibility.

The analytical derivation assumes small values of η. To assess the limitations of the derivation, Fig. S2 presents numerical data similar to that of Fig. S1, but over a broader range of η values, extending up to $\eta = 1$. Using the analytical solution, we define a

criterion for full reversal (i.e. $T_z = 1$), where $H_{peak}^2 \tau_p \geq 2\sqrt{\pi} f_{opt}/(\alpha \gamma'^2)$. This criterion is represented by the dashed curves in every panel. It is seen that complete switching occurs at slightly different values than those predicted by the criterion.

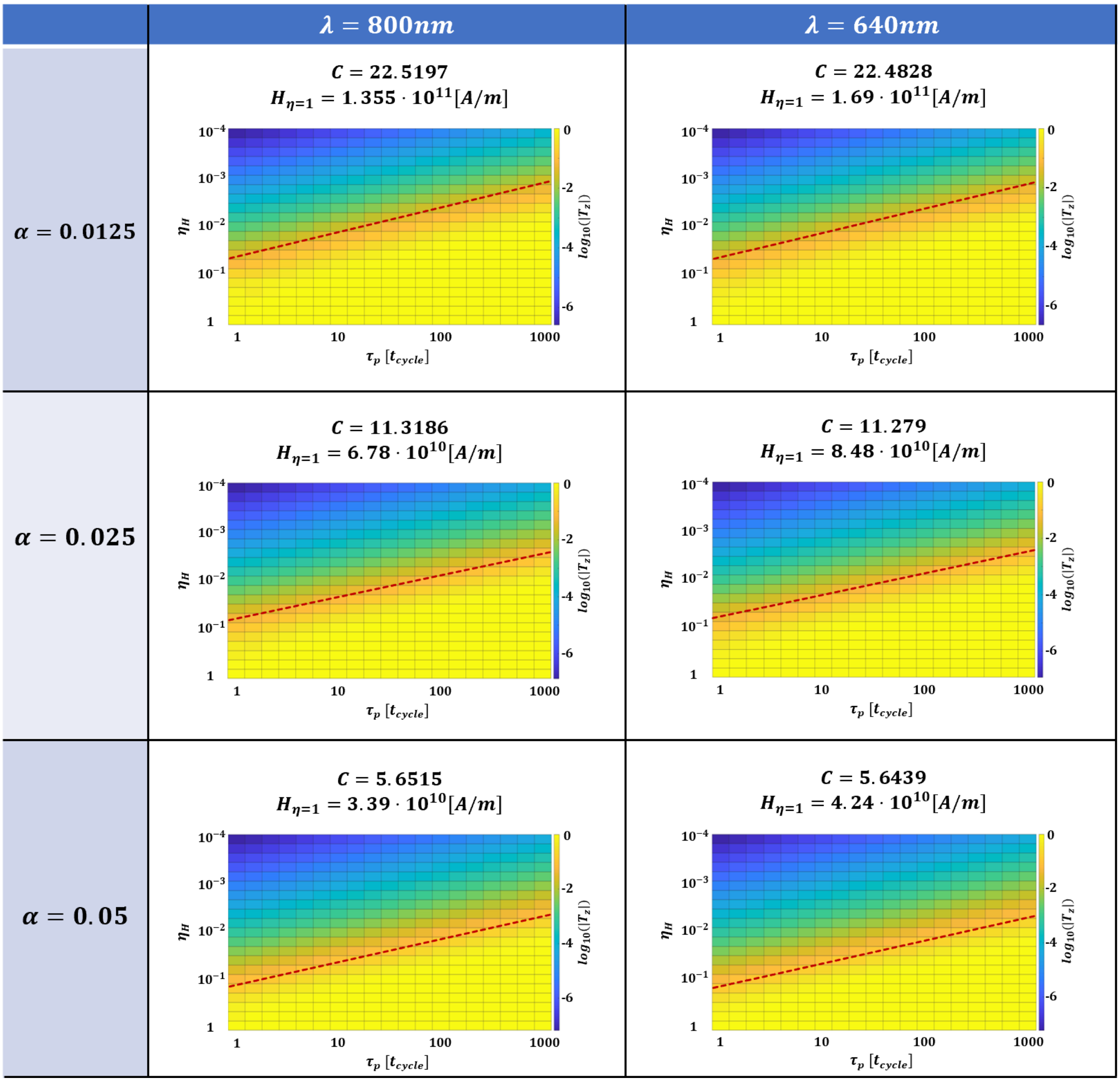

Fig. S2. $log_{10}(|T_z|)$ **after the application of an RCP optical magnetic pulse of duration** τ_p **and amplitude** $\eta_H H_{\eta=1}$**. The different panels correspond to simulated values of** $\alpha = 0.05, 0.025, 0.0125$ **and** $\lambda = 800, 640\ nm$**.** $\vec{M}$ **is initialized in the** $\hat{x}$ **direction.** $H_{\eta=1}$ **is scaled according to the relevant** α **and** f_{opt} **in each panel.** τ_p **is given in units of the optical cycle time. Data is presented on a logarithmic scale for visibility.**

In order to obtain further insight to the limit of higher η and to the limitation of the analytical solution, in Fig. S3 we plot T_z as a function of η_H on a linear scale. It is readily seen that at larger η, large-angle dynamics take place and T_z is no longer quadratic in the field amplitude. When η further increases, eventually complete switching takes place.

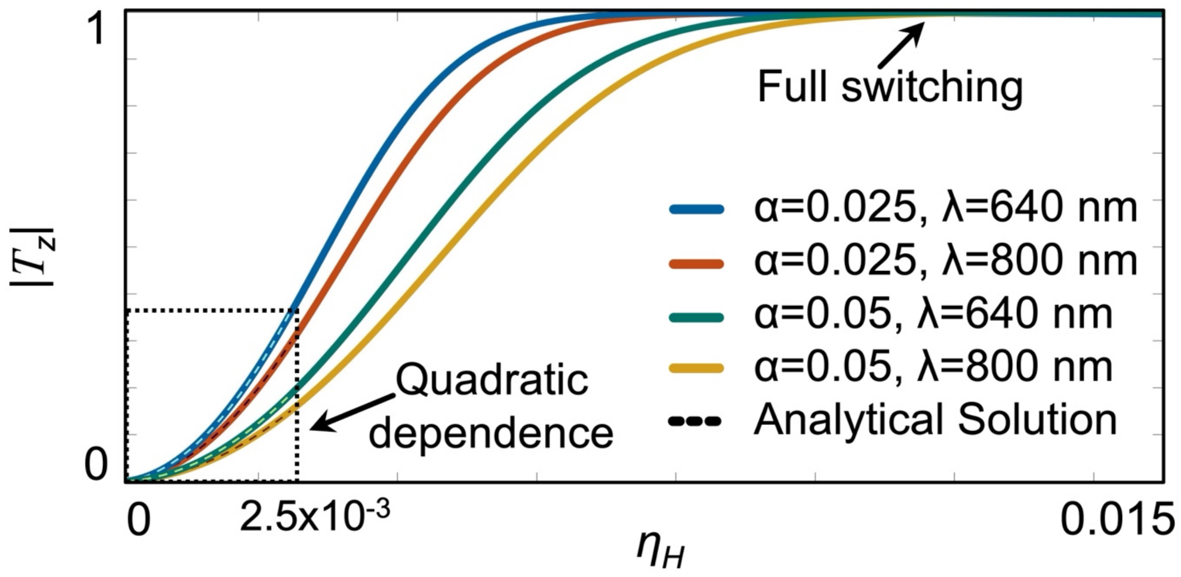

Fig. S3. Optically induced torque as a function of η_H, for $\tau_p = 12\,psec$. The analytical quadratic dependence is observed only for relatively small η_H values.

Figure S4 presents the temporal evolution of $\vec{M}$ in the limit of higher η. It is readily seen that the longitudinal torque accumulates during the interaction in this limit as well.

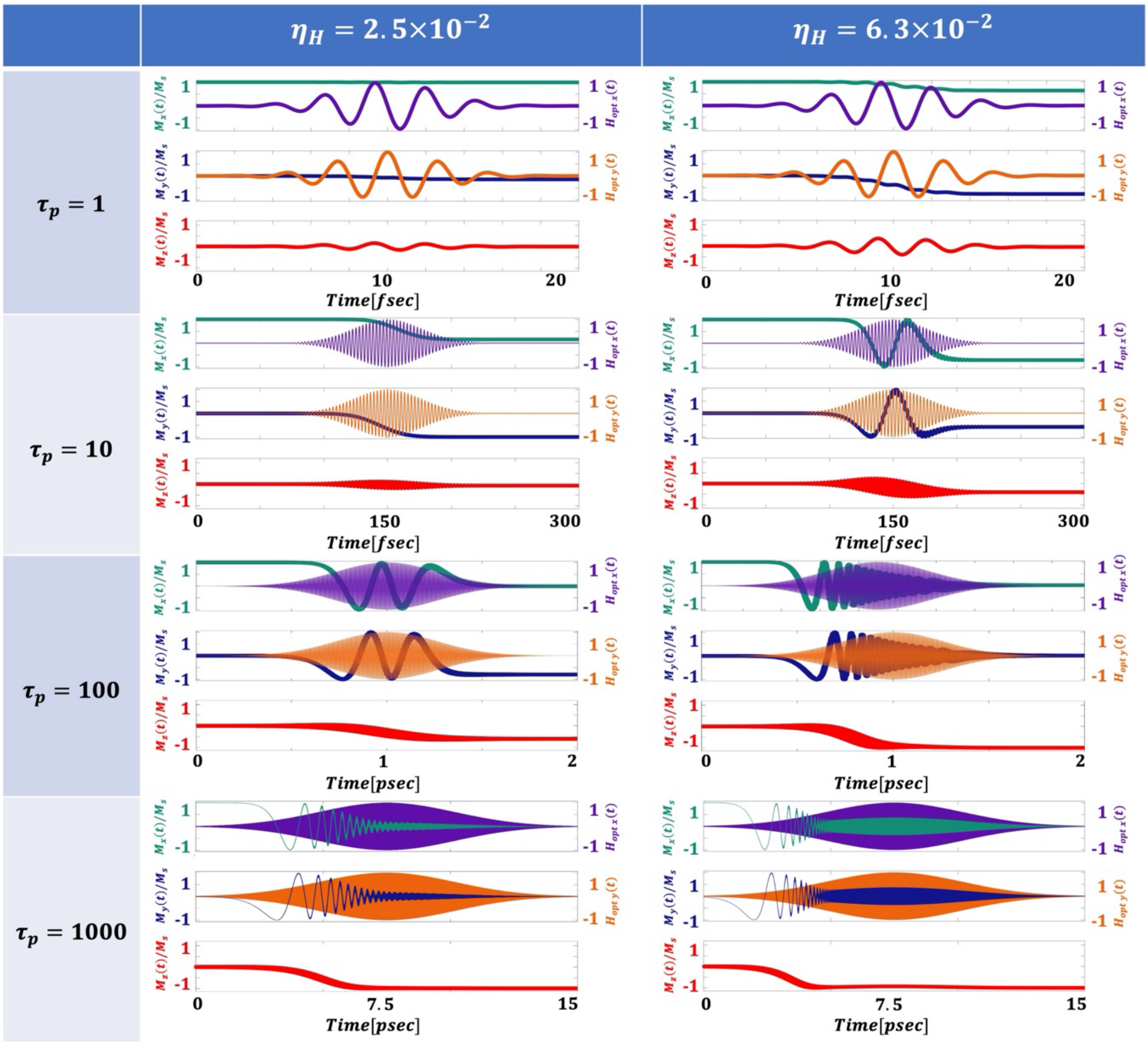

Fig. S4. In each plot, the top and middle panels depict the temporal evolution of the x and y components of $\overrightarrow{M}/M_S$ and $\overrightarrow{H}_{opt}$ in normalized units, and the bottom panel depicts M_z/M_S. H_{opt} is an $800\ nm$ RCP optical magnetic pulse of duration τ_p and amplitude $\eta_H H_{\eta=1}$, where $\eta_H = 2.5, 6.3 \times 10^{-2}, \tau_p = 1, 10, 100, 1000$. τ_p is given in units of the optical cycle time. $\alpha = 0.025$. The color code is used to highlight the optical field at shorter τ_p.

Panels (a) and (b) in Fig. S5 present the numerically calculated T_y as a function of η_H and τ_p, respectively, for the cases of $\alpha = 0.025, 0.05$ and $\lambda = 800$ and $640\ nm$. The analytical solution is represented by the dashed lines readily illustrating the agreement with the calculation.

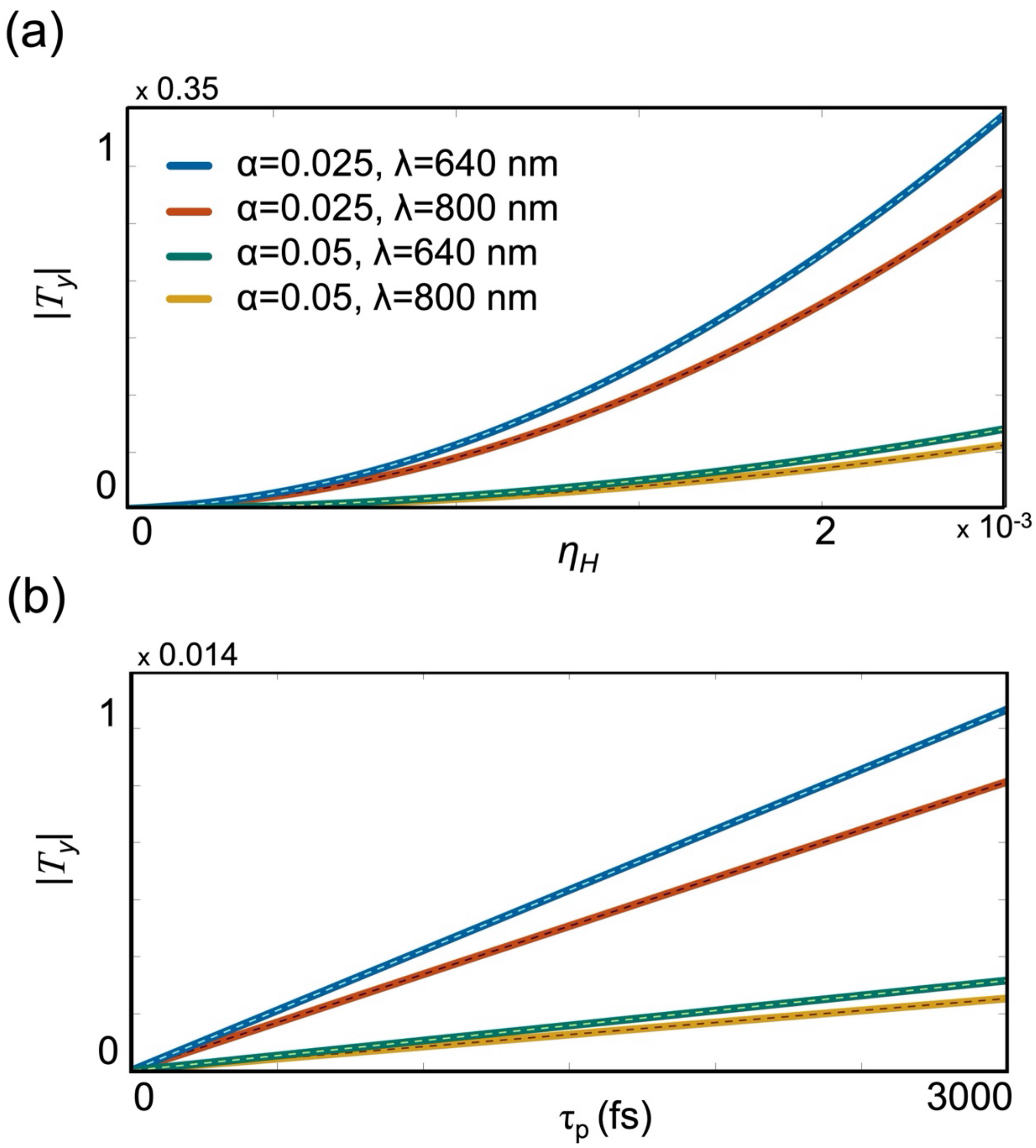

Fig. S5. (a) $|T_y|$ following the application of an RCP Gaussian magnetic pulse as a function of η_H. $\tau_p = 540\ fsec$. $\vec{M}$ is initialized in the $\hat{x}$ direction. The different curves correspond to $\alpha = 0.025, 0.05$ and $\lambda = 800, 640\ nm$. (b) $|T_y|$ after the application of an RCP Gaussian magnetic pulse as a function of τ_p, under $\eta = 2 \times 10^{-4}$. In (a) and (b), dashed lines correspond to the analytical solution.

We remark that the higher optical magnetic field amplitudes simulated in this Note ($\eta > \sim 10^{-2}$) are achievable using conventional amplified femtosecond lasers, for example by focusing a $\sim 0.5\ mJ$ pulse into a spot size of $\sim 150\ \mu m^2$. However, in practical experiments such pulses surpass the typical damage threshold of the metallic film. Nevertheless, the study of the LLG equation in this limit is instructive, as it highlights principles of the interaction, which apply also for lower η values [1].

Supplemental Note 2: Effect of anisotropy field

In this Note we examine the role of the anisotropy field on the optical torque. In our simulations (i.e. Fig. 2 of the manuscript), we considered the fluences applied in the empirical study by Choi et al. [2] where the torques are characterized quantitatively. The samples studied by Choi et al. were in-plane magnetized and primarily affected by the shape anisotropy. Therefore, we repeat the calculations for the example presented in Fig. 2(a) where the anisotropy field is given by $\vec{H}_{anis} = -M_z\hat{z}$. These calculations are presented in Fig. S6 revealing almost identical dynamics with and without $\vec{H}_{anis}$: The relative change in the $\hat{y}$ and $\hat{z}$ component of the magnetization, m_y and m_z, following the interaction is 0.7% and 0.4%, respectively.

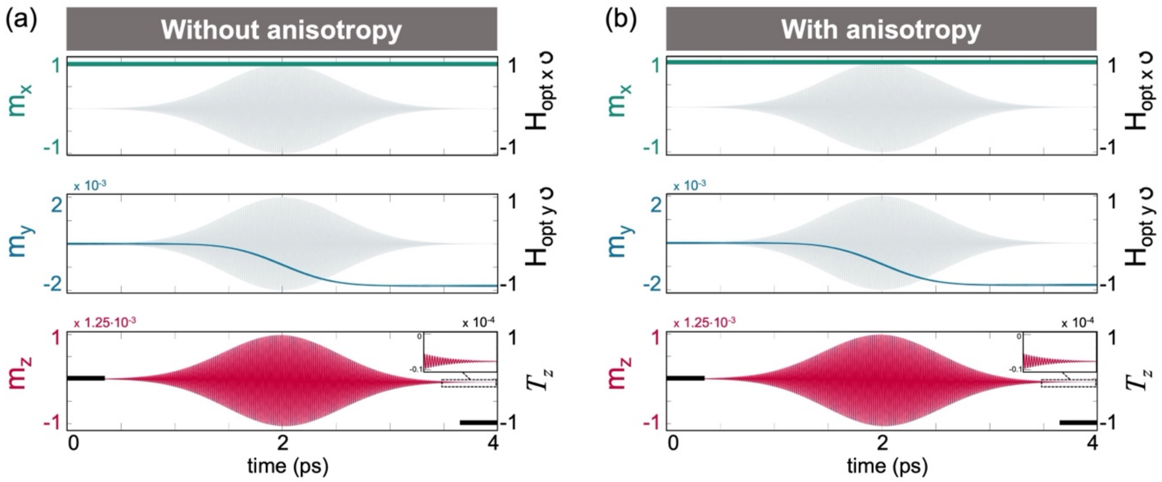

Fig. S6. Temporal evolution of $\vec{m} = \vec{M}/M_s$ induced by an RCP Gaussian pulse under $H_{peak} = 1.35 \times 10^7\ [A/m]$ (corresponding to $\eta_H = 2 \times 10^{-4}$) and $\tau_p = 540\ fsec, t_{peak} = 2\ psec$. Top and middle panels depict the temporal evolution of the x and y components of $\vec{m}$ and $\vec{H}_{opt}$ in normalized units, and the bottom panel depicts m_z. Inset: zoomed in dynamics of m_z following the pulse. The same values of $\alpha = 0.025$ and $\lambda = 800\ nm$ were used, as in Fig. 2(a) of the manuscript. (a) Without $\vec{H}_{anis}$. (b) With $\vec{H}_{anis} = -M_z\hat{z}$.

Figure S7 presents T_z as a function of H_{peak} for different durations (represented by the number of optical cycles), with and without $\vec{H}_{anis}$. Once more, the resulting torque is not significantly affected by $\vec{H}_{anis}$, as readily seen from the nearly overlapping curves.

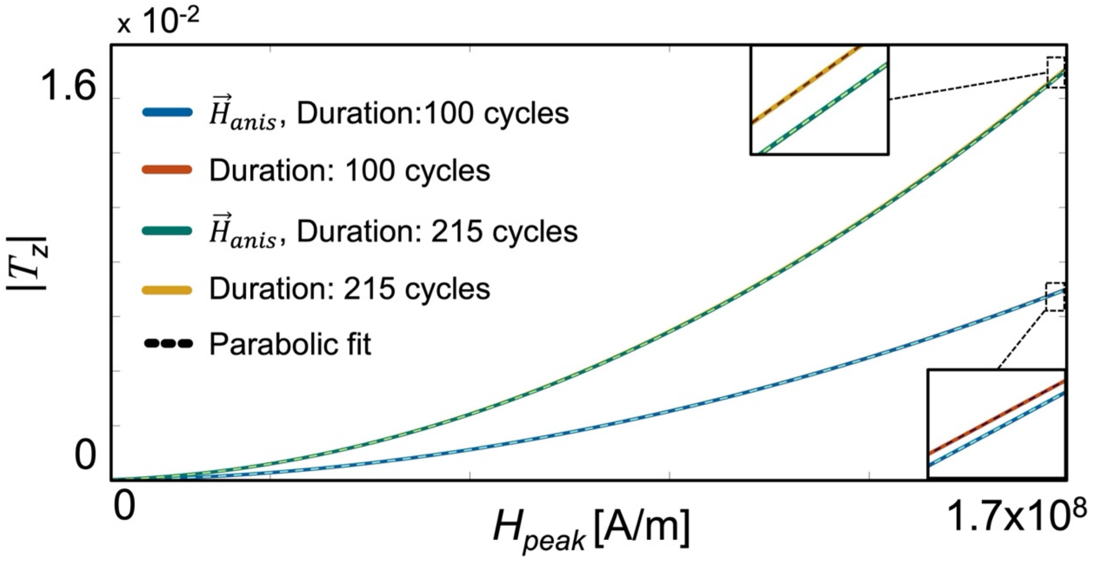

Fig. S7. $|T_z|$ **after the application of an RCP optical magnetic pulse of duration** τ_p **and amplitude** H_{peak}**, where the maximal** H_{peak} **corresponds to** $\eta = 2.5 \times 10^{-3}$. $\alpha = 0.025, \lambda = 800\, nm$. $\vec{M}$ **is initialized in the** $\hat{x}$ **direction. The different curves correspond to** $\tau_p = 100, 215$ **optical cycles, either with or without** $\vec{H}_{anis} = -M_z\hat{z}$**.**

In the general case, the shape and crystalline anisotropies can be described by the effective anisotropy field, $H_{Keff} = \left(\frac{2K_u}{\mu_0 M_s} - M_s\right)$, relevant to both in-plane and perpendicularly magnetized sample, where K_u is the out-of-plane crystalline anisotropy energy constant. Accordingly, the torque exerted by the anisotropy field is $T_{anis} \propto M_s H_{Keff} cos(\theta)\, sin(\theta)$, with θ being the polar angle of the magnetization, $\vec{M}$, in a spherical coordinate system [3]. Without any optical excitation, θ is either 0° or 90° for in-plane or perpendicularly magnetized films, respectively. Under the fluences applied in Ref. [2], the optical pulse induces only a small perturbation of $\vec{M}$. Consequently, θ remains in the vicinity of 0° or 90° and T_{anis} is relatively small.

Supplemental Note 3: Effect of longitudinal relaxation

In this Note we examine the role of the longitudinal relaxation process on the optical torque. In Fig. S8 we present simulation results which incorporate the longitudinal relaxation process, following the formalism of Ref. [4]. The figure presents the temporal evolution of $\vec{M}$ for the same parameters used in Fig. 2(a), with and without the longitudinal relaxation term, which is determined by the parameter $\alpha_{||}$. The longitudinal relaxation term is introduced to the equation of motion (Eq. (1) of the manuscript), resulting in:

$$\frac{d\vec{M}}{dt} = -\gamma' \left(\vec{M} \times \vec{H}_{opt} + \frac{\alpha}{M_s} \vec{M} \times \vec{M} \times \vec{H}_{opt} - \frac{\alpha_{||}}{M_s} (\vec{M} \cdot \vec{H}_{opt}) \vec{M} \right).$$

Assuming near-Curie temperature conditions, $\alpha_{||}$ was taken as $\alpha_{||} = \alpha$. It is readily seen that the effect of $\alpha_{||}$ is negligible in our case. More specifically, for $\eta = 2 \times 10^{-4}$, the relative change in M_x, M_y, and M_z is smaller than 10^{-5}%, 0.0625%, and 10^{-5}%, respectively. When the field amplitude is increased to $\eta = 5 \times 10^{-3}$, which is beyond typical experimental conditions, the longitudinal quenching becomes more significant and the relative change in M_x, M_y, and M_z increases to 0.3%, 0.125%, and 0.2%, respectively.

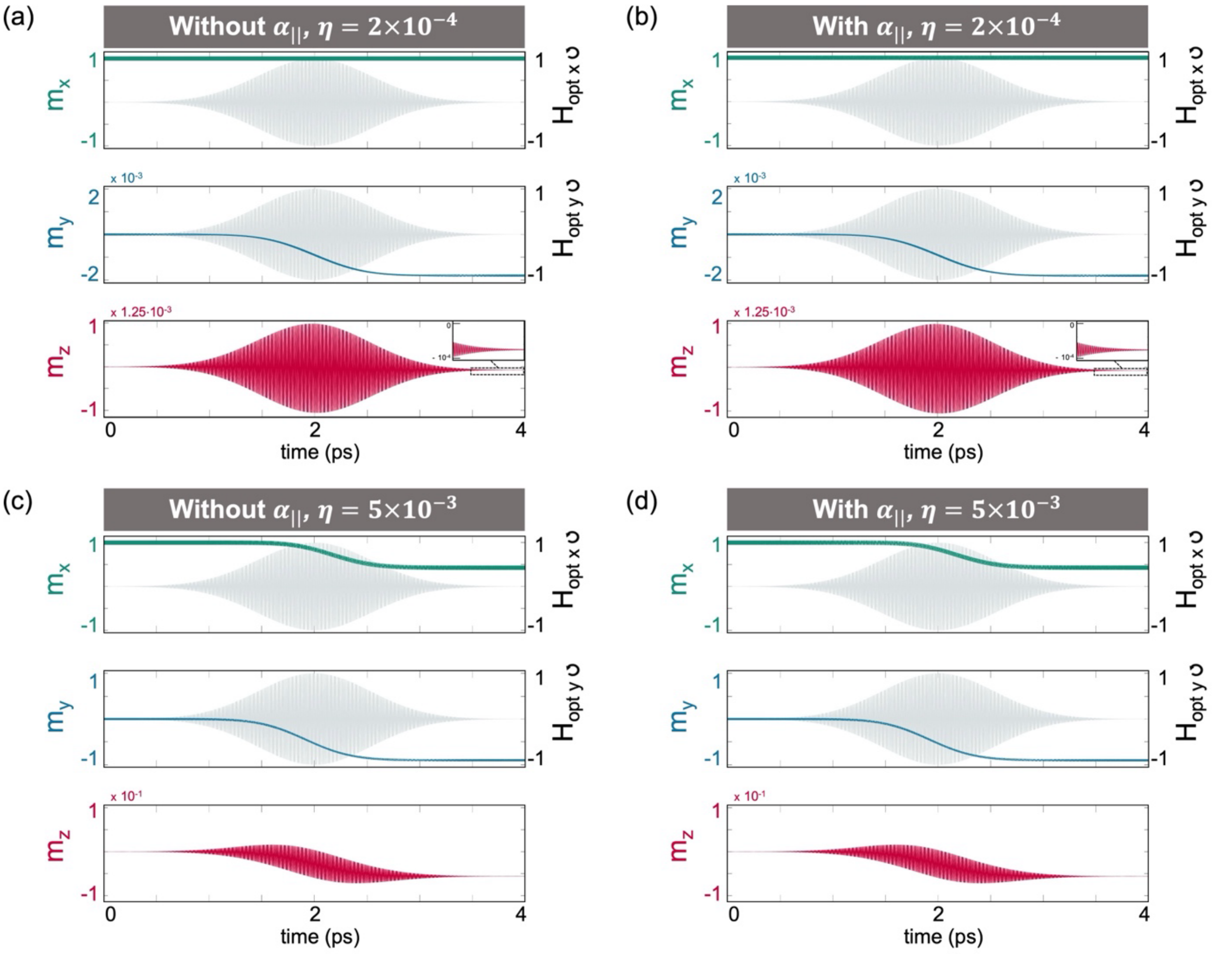

Fig. S8. Temporal evolution of $\vec{m} = \vec{M}/M_s$ induced by an RCP Gaussian pulse with and without longitudinal relaxation. In (a) and (b), $H_{peak} = 1.35 \times 10^7\ [A/m]$ corresponding to $\eta = 2 \times 10^{-4}$, and in (c) and (d) $H_{peak} = 3.4 \times 10^8\ [A/m]$ corresponding to $\eta = 5 \times 10^{-3}$. As in Fig. 2(a) from the manuscript, $\alpha = 0.025$, $\tau_p = 540\ fsec$, $t_{peak} = 2\ psec$, and $\lambda = 800\ nm$. In (a)-(d), the top and middle panels depict the temporal evolution of the x and y components of $\vec{m}$ and $\vec{H}_{opt}$ in normalized units, and the bottom panel depicts m_z. (a) and (c): calculation without $\alpha_{||}$. (b) and (d): Calculation with $\alpha_{||} = \alpha$. Inset: zoomed in dynamics of $m_z(t)$ following the pulse.

Supplemental Note 4: Dependence of $\boldsymbol{T_z}$ the polarization state

The dependence of T_z on the polarization state, $T_z \propto -sin(\phi)$, is derived analytically in Note 1 and appears in Fig. 1(d) of the manuscript. In this Note, we show analytically that $I_R - I_L \propto -sin(\phi)$, such that $T_z \propto I_R - I_L$.

We consider an optical magnetic field of the form:

$$\vec{H}_{opt\ \phi}(t) = H_{peak} \begin{pmatrix} cos(\omega_{opt} t) \\ cos(\omega_{opt} t - \phi) \\ 0 \end{pmatrix} e^{-\frac{(t-t_{peak})^2}{2\tau_p^2}}. \quad (S4.1)$$

In order to decompose $\vec{H}_{opt\ \phi}(t)$ into its RCP and LCP components, we use the $\hat{R}$ and $\hat{L}$ operators:

$$\hat{R} = \frac{1}{\sqrt{2}} \begin{pmatrix} 1 \\ j \\ 0 \end{pmatrix}, \quad \hat{L} = \frac{1}{\sqrt{2}} \begin{pmatrix} 1 \\ -j \\ 0 \end{pmatrix} \quad (S4.2)$$

Applying $\hat{R}$ and $\hat{L}$ on $\vec{H}_{opt\ \phi}(t)$, we obtain:

$$H_{\hat{R}} = \vec{H}_{opt\ \phi}(t) \cdot \hat{R} = \frac{H_{peak}}{\sqrt{2}} \left(cos(\omega_{opt} t) - sin(\omega_{opt} t - \phi)\right) e^{-\frac{(t-t_{peak})^2}{2\tau_p^2}} \quad (S4.3)$$

$$H_{\hat{L}} = \vec{H}_{opt\ \phi}(t) \cdot \hat{L} = \frac{H_{peak}}{\sqrt{2}} \left(cos(\omega_{opt} t) + sin(\omega_{opt} t - \phi)\right) e^{-\frac{(t-t_{peak})^2}{2\tau_p^2}} \quad (S4.4)$$

The intensity difference between the two components is given by:

$$I_{\hat{R}} - I_{\hat{L}} = \frac{1}{2} \frac{c\epsilon_0}{Z_0} \left(H_{\hat{R}}^2 - H_{\hat{L}}^2\right), \quad (S4.5)$$

where c, ϵ_0, and Z_0 are the speed of light, vacuum permittivity, and the impedance of free space, respectively. Substituting Eq. (S4.3) and (S4.4) into (S4.5) we obtain:

$$I_{\hat{R}} - I_{\hat{L}} = \frac{1}{2} \frac{c\epsilon_0}{Z_0} \frac{H_{peak}^2}{2} \left(-4\, cos(\omega_{opt} t)\, sin(\omega_{opt} t - \phi)\right) e^{-\frac{(t-t_{peak})^2}{\tau_p^2}}. \quad (S4.6)$$

Finally, we obtain:

$$I_{\hat{R}} - I_{\hat{L}} = -\frac{c\epsilon_0}{Z_0} H_{peak}^2 \left(sin(2\omega_{opt} t - \phi) + sin(\phi)\right) e^{-\frac{(t-t_{peak})^2}{\tau_p^2}}. \quad (S4.7)$$

The rapid-oscillating part averages to zero whereas the rectified part multiplying the Gaussian envelope averages to a finite quantity. Hence, we conclude that $< I_{\hat{R}} - I_{\hat{L}} >$ $\propto -sin(\phi)$, as shown in Fig. 1(d) of the manuscript.

Supplemental Note 5: Interaction with linearly polarized pulses

Figure S9 presents the temporal evolution of the $\vec{M}$ under a linearly polarized magnetic pulse that is polarized either in the $\hat{y}$ or $\hat{x}$-$\hat{y}$ directions for $\eta_H = 10^{-3}$ and 10^{-2}, and $\tau_p = 200$ and $500 \times t_{cycle}$. It is seen that for low powers the $\hat{x}$ component of $\vec{M}$ is nearly unaffected and the $\hat{y}$ component of $\vec{M}$ is nearly independent of the $\hat{y}$ or $\hat{x}$-$\hat{y}$ polarization direction. On the other hand, for the higher amplitudes, the $\hat{x}$ component of $\vec{M}$ significantly evolves, where the evolution of the $\hat{y}$ component depends on the polarization direction. Although no net torque is induced at the end of the interaction in either low-power or high-power limits or polarization states, $\vec{M}$ undergoes non-trivial dynamics during the interaction with the pulse. The figure also reveals that the pulse duration does not affect the overall behavior: It is seen that the peak amplitude of the different components of $\vec{M}$ reach the same values for different τ_p as long as η_H remains fixed.

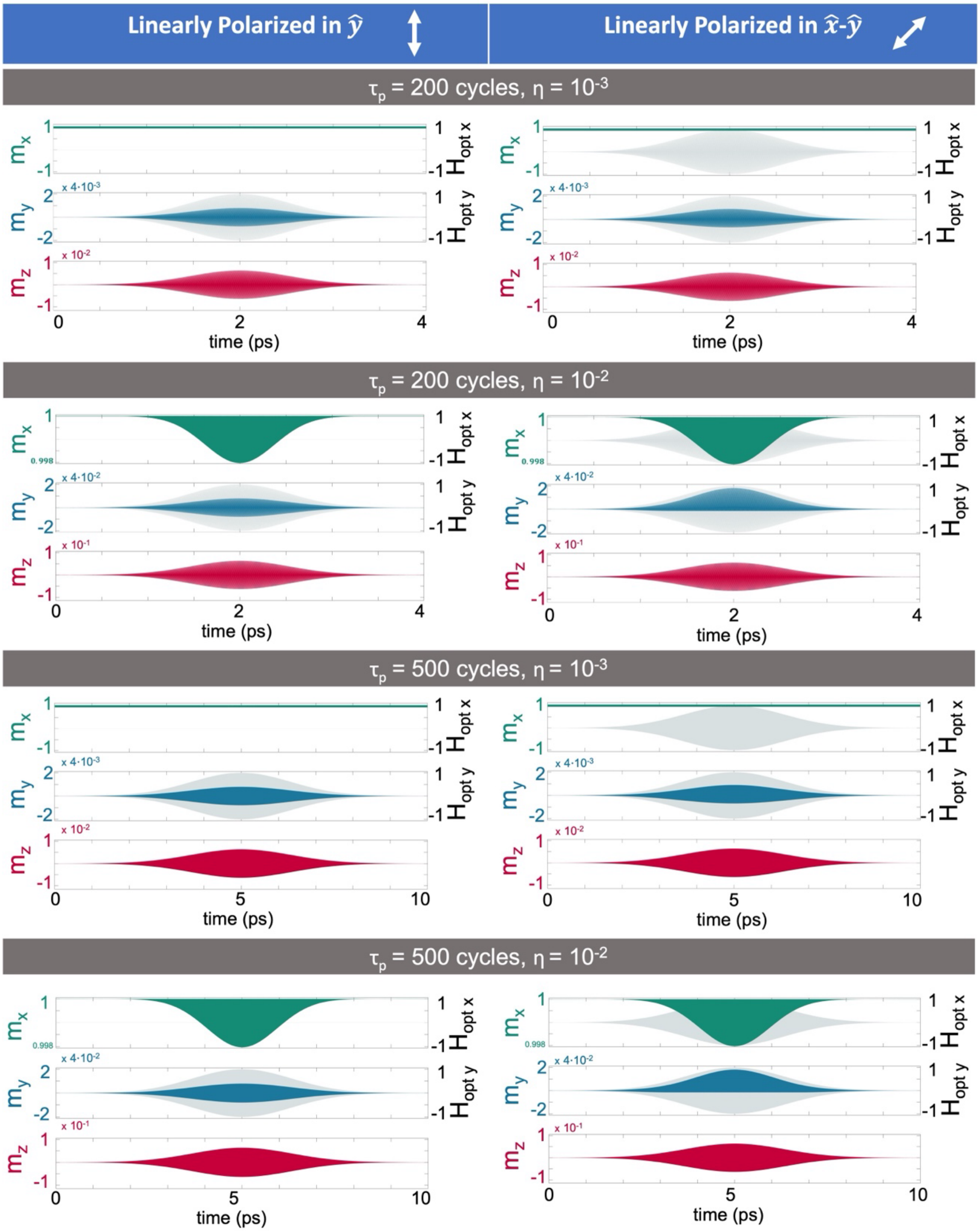

Fig. S9. Evolution of the normalized magnetization, $\vec{m} = \vec{M}/M_S$, under an LP $\vec{H}_{opt}$ pulse polarized along the $\hat{y}$ and $\hat{x}$-$\hat{y}$ direction. First, second, and third panels in each plot depicts the evolution of the $\hat{x}$, $\hat{y}$, and $\hat{z}$ component of $\vec{m}$ and $\vec{H}_{opt}$. H_{opt} is an $800\ nm$ LP optical magnetic pulse of duration τ_p and amplitude determined by η_H. $\vec{M}$ is initialized in the $\hat{x}$ direction, and H_{opt} is linearly polarized along the $\hat{y}$ and $\hat{x}$-$\hat{y}$ direction. $M_S = 3 \times 10^5\ A/m$, $\alpha = 0.025$.

Supplemental Note 6: Multi-pulse regime

In this Note, we demonstrate that T_y and T_z induced by a single pulse can be equivalently induced by multiple pulses, provided that their total fluence matches that of the single pulse. In Fig. S10 we present T_z for multiple RCP optical magnetic pulses as a function of amplitude for different values of α and λ. The data is displayed in the same manner as in Fig. S2. The duration of each pulse is $\tau_p = 10\ fsec$. Dashed curves correspond to the complete switching criterion $\#pulses \times H_{peak}^2 \tau_p = 2\sqrt{\pi} f_{opt} / (\alpha {\gamma'}^2)$ in every panel. As in Fig. S2, it is seen that the complete switching occurs at slightly different values than those predicted by the criterion.

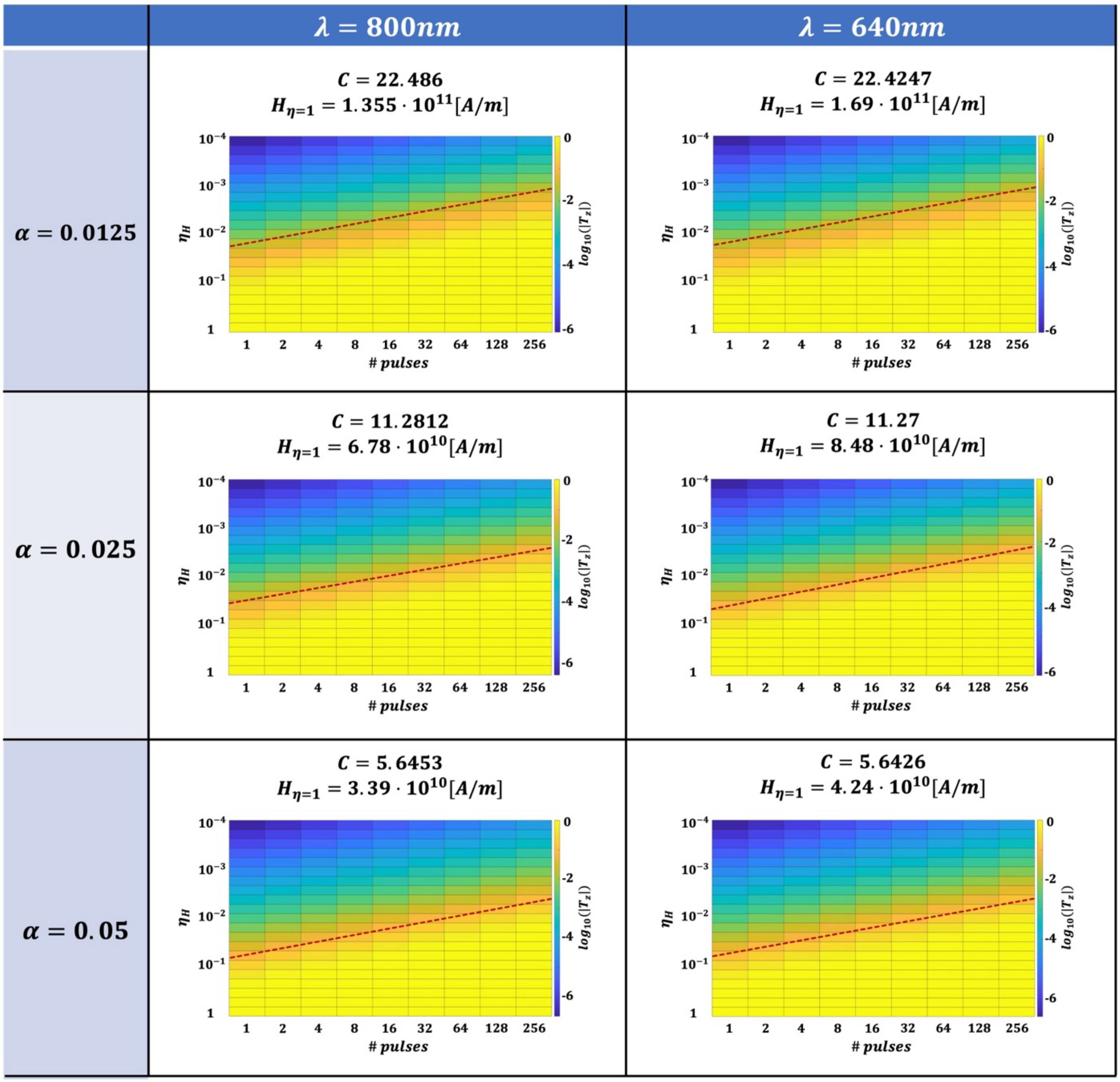

Fig. S10. $\boldsymbol{log_{10}(|T_z|)}$ **after the application of** $\boldsymbol{\#pulses}$ **RCP optical magnetic pulses of duration** $\boldsymbol{\tau_p = 10\ fsec}$ **and amplitude** $\boldsymbol{\eta_H H_{\eta=1}}$**. The different panels correspond**

to simulated values of $\alpha = 0.05, 0.025, 0.0125$ and $\lambda = 800, 640\ nm$. $H_{\eta=1}$ is scaled according to the relevant α and f_{opt} in each panel. $\vec{M}$ is initialized in the $\hat{x}$ direction. Data is presented on a logarithmic scale for visibility.

Figure S11 presents the temporal evolution of $\vec{M}$ in the multi-pulse regime in the limit of higher η. The figure presents temporal plots of simulations with $\tau_p = 10\ fsec$, $\eta_H = 1, 2.5 \times 10^{-2}$ and $\#pulses = 2, 10, 50, 200$, where $\alpha = 0.025$ and $\lambda = 800\ nm$. Comparison with Fig. S4 reveals that the outcome of the interaction in the multi- and single- pulse regimes is similar.

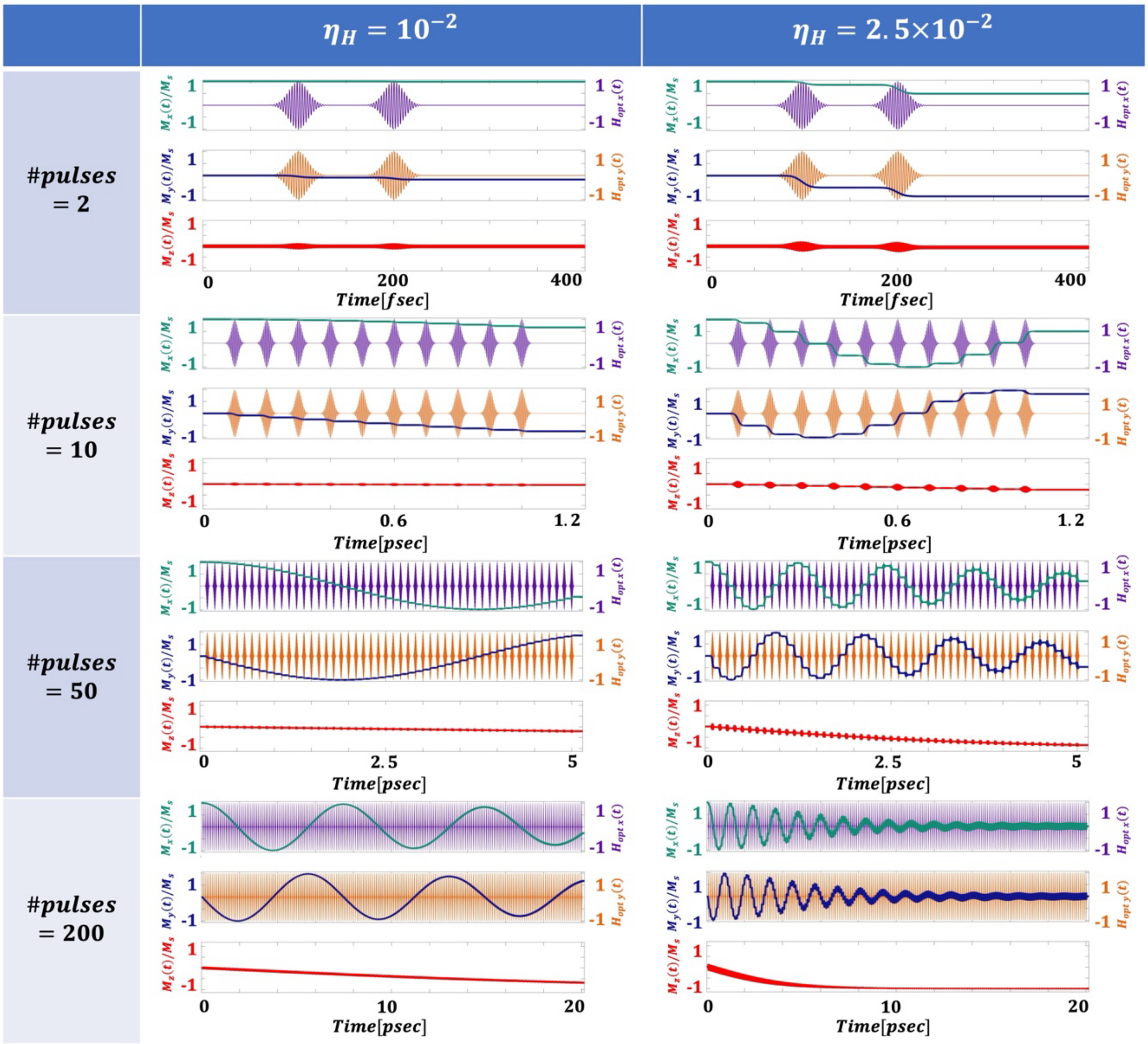

Fig. S11. In each plot, the top and middle panels depict the temporal evolution of the x and y components of $\vec{M}/M_S$ and $\vec{H}_{opt}$ in normalized units, and the bottom panel depicts M_z/M_S. H_{opt} is an $800\ nm$ RCP optical magnetic pulse of duration $\tau_p = 10\ fsec$ and amplitude $\eta_H H_{\eta=1}$. $\alpha = 0.025$, $\eta_H = 1, 2.5 \times 10^{-2}$, $\#pulses = 2, 10, 50, 200$. The color code is used to highlight the optical field at smaller $\#pulses$.

Supplemental Note 7: CW regime

In this Note we show that T_y and T_z induced by CW excitations follow the same principles demonstrated for pulsed excitations. In Fig. S12 we plot T_z after the application of a CW RCP optical beam, for the same parameters used in Fig. 1(b) and (c) in the main text. In Fig. S12(a) we simulate $\vec{H}_{opt\,CW}(t)$ for $10^{-4} \leq \eta_H \leq 2.5 \times 10^{-3}$ and for $t_{CW} = 20\,ps$, under $\alpha = 0.025, 0.05$ and $\lambda = 800, 640\,nm$. The analytical solution in this case is $T_z = \frac{1}{2\pi\alpha}\frac{t_{CW}}{t_{cycle}}\eta^2$ stemming from Eq. (S1.9) and is represented by the dashed lines, readily illustrating the agreement with the numerical calculation. In Fig. S12(b) we simulate $\vec{H}_{opt\,CW}(t)$ for a fixed $\eta = 10^{-3}$ and $0 \leq t_{CW} \leq 100\,ps$. The agreement with the analytical solution is seen as well.

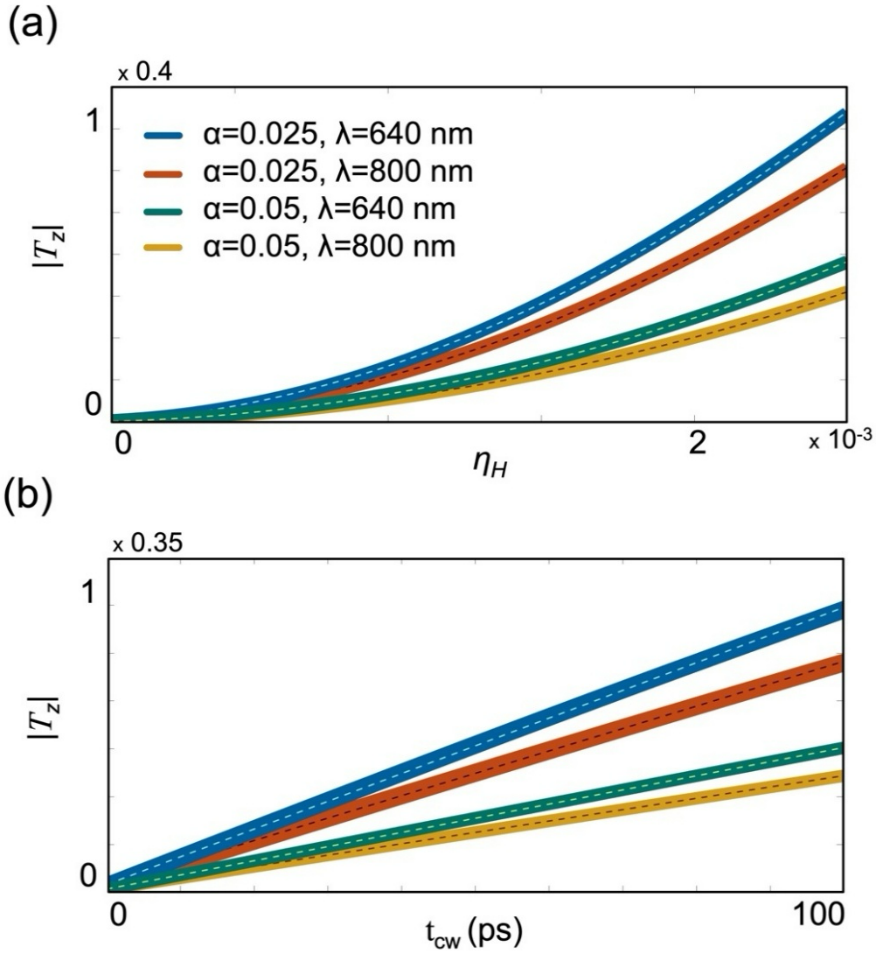

Fig. S12. (a) Optically induced torque induced by a CW RCP magnetic field as a function of η_H, for $t_{CW} = 20\,psec$. The different curves correspond to $\alpha = 0.025, 0.05$ and $\lambda = 800, 640\,nm$. (b) Optically induced torque as a function of t_{CW}, for $\eta = 10^{-3}$. In (a) and (b), dashes lines correspond to the analytical solution.

Supplemental Note 8: Derivation of V_{LLG}^{FE} and V_{LLG}^{IFE}

To derive V_{LLG}^{FE} we consider the linearized LLG equation [5]:

$$\frac{d\vec{m}}{dt} + \gamma\mu_0\vec{m}\times\vec{H}_{DC} + \alpha\frac{d\vec{m}}{dt}\times\vec{m} = -\gamma\mu_0 M_S\vec{m}\times\vec{h} \qquad (S8.1)$$

Under steady state conditions, we take:

$$\vec{m} = me^{j\omega t}\begin{pmatrix}1\\ \mp j\\ 0\end{pmatrix}, \vec{h} = he^{j\omega t}\begin{pmatrix}1\\ \mp j\\ 0\end{pmatrix}, \vec{H}_{DC} = \begin{pmatrix}0\\ 0\\ H_{DC}\end{pmatrix}, \qquad (S8.2)$$

where $\mp$ correspond to RCP/LCP states, respectively.

Rearranging Eq. (S8.1) into component form we obtain:

$$j\omega m\begin{pmatrix}m_x\\ m_y\\ 0\end{pmatrix} + \gamma\mu_0 H_{DC} m\begin{pmatrix}m_y\\ -m_x\\ 0\end{pmatrix} + j\omega\alpha\begin{pmatrix}m_y\\ -m_x\\ 0\end{pmatrix} = -\gamma\mu_0 M_S h\begin{pmatrix}h_y\\ -h_x\\ 0\end{pmatrix}. \qquad (S8.3)$$

We define the small-signal magnetic susceptibility as $\chi = m/h$ which results in:

$$\chi_{RCP} = \frac{-\gamma\mu_0 M_S}{\omega - \gamma\mu_0 H_{DC} - j\omega\alpha}, \qquad \chi_{LCP} = \frac{\gamma\mu_0 M_S}{\omega + \gamma\mu_0 H_{DC} + j\omega\alpha}. \qquad (S8.4)$$

It is readily seen that χ_{RCP} displays resonant behavior near $\omega = \gamma\mu_0 H_{DC}$, while χ_{LCP} doesn't. This is due to the precession direction dictated by H_{DC}. The Faraday rotation angle, Θ_{FE}, is given by the difference in the propagation vectors of RCP and LCP light multiplied by the optical length:

$$\Theta_{FE} = \frac{1}{2}(k_{RCP} - k_{LCP})L. \qquad (S8.5)$$

Substituting $k = \frac{\omega n}{c} = \frac{\omega\sqrt{\epsilon_r\mu_r}}{c} = \frac{\omega\sqrt{\epsilon_r(1+\chi)}}{c}$, Θ_{FE} becomes:

$$\Theta_{FE} = \frac{1}{2}\frac{\omega}{c}\sqrt{\epsilon_r}\left(\sqrt{1+\chi_{RCP}} - \sqrt{1+\chi_{LCP}}\right)L, \qquad (S8.6)$$

which for χ_{RCP} and χ_{LCP} takes the form:

$$\Theta_{FE} = \frac{1}{2}\frac{\omega}{c}\sqrt{\epsilon_r}\left(\sqrt{1 - \frac{\gamma\mu_0 M_S}{\omega - \gamma H_0 - j\omega\alpha}} - \sqrt{1 + \frac{\gamma\mu_0 M_S}{\omega + \gamma H_0 + j\omega\alpha}}\right)L. \qquad (S8.7)$$

Θ_{FE} is evaluated away from resonance, since at optical frequencies $\omega \gg \gamma H_{DC}$, while also $\omega \gg \gamma M_S$, therefore:

$$\Theta_{FE} = -\frac{1}{2}\frac{\gamma\mu_0 M_S}{1+\alpha^2}\frac{\sqrt{\epsilon_r}}{c}L. \qquad (S8.8)$$

The Faraday rotation angle is commonly expressed in terms of the Verdet constant, according to:

$$\Theta_{FE} = V\mu_0 H_{DC} L. \qquad (S8.9)$$

In order to obtain an expression of the same form, we substitute $M_S = \mu_0 \chi_{DC} H_{DC}$ and find:

$$V_{LLG}^{FE} = -\frac{1}{2}\frac{\gamma\mu_0\chi_{DC}}{1+\alpha^2}\frac{\sqrt{\epsilon_r}}{c}. \qquad (S8.10)$$

Lastly, we turn to show that the reciprocity between the FE and IFE breaks down also within the framework of the LLG equation (namely, $V_{LLG}^{FE} \neq V_{LLG}^{IFE}$). In order to calculate V_{LLG}^{IFE}, we use the relation [6]:

$$M = \frac{I_{RCP} - I_{LCP}}{2c\pi}\lambda V, \qquad (S8.11)$$

where V is the static Verdet constant of the material. The optical torque derived from the LLG equation is of the form:

$$\frac{M_z}{M_s} = \frac{\alpha\gamma^2}{2\sqrt{\pi} f_{opt}(1+\alpha^2)^2} H_{peak}^2 \tau_p. \qquad (S8.12)$$

By comparing Eq. (S8.11) and (S8.12), we obtain:

$$V_{LLG}^{IFE} = M_S \frac{\sqrt{\pi}}{c}\frac{\alpha}{(1+\alpha^2)^2}\gamma^2\mu_0\tau_p. \qquad (S8.13)$$

Equation (S8.13) clearly differs from Eq. (S8.10), demonstrating that the FE and IFE yield fundamentally different Verdet constants.

References

[1] B. Assouline, A. Capua, Helicity-dependent optical control of the magnetization state emerging from the Landau-Lifshitz-Gilbert equation, Physical Review Research, 6 (2024) 013012.
[2] G.-M. Choi, A. Schleife, D.G. Cahill, Optical-helicity-driven magnetization dynamics in metallic ferromagnets, Nature Communications, 8 (2017) 15085.
[3] A. Capua, S.-H. Yang, T. Phung, S.S.P. Parkin, Determination of intrinsic damping of perpendicularly magnetized ultrathin films from time-resolved precessional magnetization measurements, Physical Review B, 92 (2015) 224402.
[4] I. Korniienko, P. Nieves, O. Chubykalo-Fesenko, D. Legut, Magnetization dynamics induced by ultrashort terahertz radiation: Toward designing spin-based terahertz sensors, Physical Review Applied, 21 (2024) 014025.
[5] Alexander G. Gurevich, G.A. Melkov, Magnetization Oscillations and Waves, CRC Press, Boca Raton Florida, 1996.
[6] P.S. Pershan, J.P. van der Ziel, L.D. Malmstrom, Theoretical Discussion of the Inverse Faraday Effect, Raman Scattering, and Related Phenomena, Physical Review, 143 (1966) 574-583.
